# The MdHB7L–MdICE1L–MdHOS1 Module Fine‐Tunes Apple Cold Response via CBF‐Dependent and CBF‐Independent Pathways

**DOI:** 10.1002/advs.202501524

**Published:** 2025-04-26

**Authors:** Jie Yang, Na Li, Ming Li, Ran Yi, Lina Qiu, Kangning Wang, Shuang Zhao, Fengwang Ma, Ke Mao

**Affiliations:** ^1^ State Key Laboratory for Crop Stress Resistance and High‐Efficiency Production/Shaanxi Key Laboratory of Apple College of Horticulture Northwest A & F University Yangling Shaanxi 712100 China

**Keywords:** apple, cold response, competitive interaction, HD‐Zip transcription factor, protein degradation

## Abstract

Cold stress is a major environmental factor limiting crop yield, quality, and geographical distribution worldwide. The homeodomain‐leucine zipper (HD‐Zip) transcription factor (TF) family plays a role in regulating plant abiotic stress responses, but the underlying mechanisms remain unclear. A HD‐Zip TF, MdHB7L, is identified as promoting cold tolerance in apple. MdHB7L interacts with MdICE1L, enhancing its transcriptional activation of MdCBFs, and directly binds to MdCBF promoters to activate their expression. Conversely, MdICE1L inhibits the direct binding of MdHB7L on MdCBF promoters, revealing that MdHB7L acts as a cofactor rather than a TF when interacting with MdICE1L. Using ChIP‐seq and RNA‐seq, MdHB7L is found to directly regulate the expression of several key genes involved in ROS scavenging and biosynthesis of anthocyanins, soluble sugars, and proline, thereby enhancing apple cold tolerance. The E3 ubiquitin ligase MdHOS1 negatively regulates cold tolerance by interacting with and mediating the degradation of MdHB7L and MdICE1L, with a preference for MdICE1L over MdHB7L. This preference inhibits the MdHOS1–MdHB7L interaction and stabilizes MdHB7L, allowing it to sustain the plant's cold response as a TF after MdICE1L degradation. These findings provide new insights into the dynamic plant response to cold stress mediated by the MdHB7L–MdICE1–MdHOS1 module.

## Graphical abstract

In early cold response, MdICE1L utilizes MdHB7L as a cofactor to facilitate the transcriptional activation of MdCBFs, thereby activating cold signaling rapidly and strongly. Subsequently, MdICE1L is degraded by MdHOS1. Meanwhile, MdHB7L is released and accumulates. It then acts as a TF to directly regulate the expression of MdCBFs and other target genes, sustaining the plant's response to cold environments.

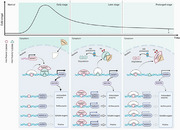

## 1 Introduction

Low temperature is a critical environmental factor that impacts plant growth and development. It limits plants' geographical distribution and negatively affects crop yield and quality.^[^ ^1^, ^2^ ^]^ Cold stress in plants is categorized into two types based on temperature ranges and the physiological mechanisms involved: chilling stress (0–15 °C) and freezing stress (< 0 °C).^[^ ^2^, ^3^ ^]^ Most temperate plants can enhance their freezing tolerance through a process called cold acclimation, where they are first exposed to low, non‐freezing temperatures.^[^ ^2^ ^]^ Sudden frost can be particularly damaging to plants.^[^ ^4^ ^]^ Cold stress can damage cell membrane structures, leading to decreased extracellular water potential, increased ion leakage, and higher malondialdehyde (MDA) content. It also triggers the overproduction of reactive oxygen species (ROS).^[^ ^5^, ^6^ ^]^ To withstand cold stress, plants have developed complex mechanisms that involve alterations in physiological and biochemical processes. These changes are accompanied by the modified expression of thousands of genes.^[^ ^7^, ^8^ ^]^

Previous studies in *Arabidopsis* have shown that the C‐repeat binding factor/ dehydration‐responsive element binding protein 1(CBF/DREB1)–cold responsive genes (CORs) signaling pathway is crucial for cold response.^[^ ^8^, ^9^ ^]^ Low temperatures activate CBFs, which then bind to DRE/C‐repeat (CRT) elements in the promoters of specific CORs. These include COR15A/B, COR47, RD29A/B, and KIN1, triggering their transcription.^[^ ^5^, ^7^, ^9^ ^]^ The upregulation of CORs leads to various physiological and biochemical changes that enhance plant cold resistance. These changes include the accumulation of osmoregulatory substances like soluble sugars, soluble proteins, and proline, increased antioxidant enzyme activity for excessive ROS scavenging, and the buildup of secondary metabolites such as ascorbic acid, carotenoids, flavonoids, and anthocyanins, which mitigate oxidative damage through non‐enzymatic systems.^[^ ^6^, ^10^ ^]^ The beneficial roles of CBF orthologues in CORs expression and cold tolerance have been observed in numerous other plant species, including rice,^[^ ^11^ ^]^ wheat,^[^ ^12^ ^]^ maize,^[^ ^13^ ^]^ tomato,^[^ ^14^ ^]^ poplar,^[^ ^15^ ^]^ and apple.^[^ ^16^ ^]^ This indicates that the CBF–COR pathway is highly conserved across plants.

The crucial role of CBFs in cold response has led to the identification of various transcription factors (TFs) that directly regulate their expression under cold conditions. These include bHLHs, MYBs, ERFs, NACs, bZIPs, and BBXs.^[^ ^6^ ^]^ Among these, inducer of CBF expression 1 (ICE1), a bHLH family member, is a well‐known positive regulator of plant cold response.^[^ ^2^ ^]^ In *Arabidopsis*, cold exposure rapidly activates ICE1, which then binds to the E‐box/MYC *cis*‐elements (CANNTG) in the CBF3/DREB1A promoter to activate expression.^[^ ^2^, ^9^ ^]^ ICE1 homologs in other plants, such as SlICE1a in tomato,^[^ ^17^ ^]^ PuICE1 in pear,^[^ ^18^ ^]^ and MdCIbHLH1 and MdMdICE1L in apple,^[^ ^16^, ^17^, ^19^, ^20^ ^]^ share similar functions in regulating CBF expression and cold tolerance. Given ICE1's key role in cold signaling, several TFs that interact with ICE1 to regulate CBF transcription have been identified, such as the positive regulator CDC5 and the negative regulators MYB15, MYC67, and MYC70 in *Arabidopsis*.^[^ ^21^, ^22^, ^23^ ^]^ Further studies have shown that these TFs can also regulate CBF expression independently of ICE1 by directly binding to CBF promoters. In apple, TFs like MdBBX37 and MdbHLH4 regulate CBF transcription in both ICE1‐dependent and ICE1‐independent manners.^[^ ^16^, ^20^ ^]^ Moreover, some TFs can regulate cold tolerance through both CBF‐dependent and CBF‐independent pathways. For instance, MdNAC104 in apple directly activates MdCBFs expression, as well as the expression of anthocyanin synthesis genes and antioxidant enzyme‐coding genes, thereby enhacing cold tolerance through multiple ways.^[^ ^6^ ^]^ This illustrates the complexity of TF regulation in plant cold response.

The homeodomain‐leucine zipper (HD‐Zip) TF family is unique to plants and plays a crucial role in regulating growth, development, and biotic and abiotic stress responses.^[^ ^24^, ^25^, ^26^ ^]^ These TFs feature highly conserved HD and Zip domains and are classified into four subgroups (I–IV). Among these, HD‐Zip I members are involved in cold response.^[^ ^24^, ^27^ ^]^ For instance, overexpressing the cold‐responsive AtHB13 or its sunflower homologue HaHB1 enhances cold tolerance in *Arabidopsis*.^[^ ^28^ ^]^ Similarly, overexpressing TaHDZipI‐5 in wheat boosts tolerance to drought and freezing stress, although it negatively impacts biomass and yield.^[^ ^29^ ^]^ HD‐Zip TFs regulate gene expression by binding to specific promoter sequences. HD‐Zip I TFs preferentially bind to the pseudo‐palindromic sequence “CAAT(A/T)ATTG”, as identified through high‐affinity binding site selection from random DNA sequences.^[^ ^30^, ^31^ ^]^ Additionally, sequences like “TAATTA/G”, “GAATNATTC”, “TAATNATTA”, and “AAATNATTT” with varying lengths may serve as consensus binding sites for HD proteins.^[^ ^32^ ^]^ Our previous research has shown that the HD‐Zip I TFs MdHB7 and MdHB7‐like (MdHB7L) positively regulate drought and salt stress responses in apples.^[^ ^33^, ^34^, ^35^, ^36^ ^]^ However, the role of HD‐Zip TFs in apple cold response remains unexplored, and the underlying mechanisms of how HD‐Zip TFs mediated plant abiotic stress responses are largely unknown.

Post‐translational modifications, including phosphorylation, sumoylation, and ubiquitination, influence the stability, transcriptional activity, and protein–protein interactions of various TFs, thereby playing a crucial role in regulating plant growth and stress responses.^[^ ^2^, ^37^ ^]^ For instance, the transcriptional activity and protein stability of ICE1 are controlled by multiple post‐translational modifications. When exposed to cold, the SnRK2 kinase OST1 is rapidly activated. It then interacts with and phosphorylates ICE1, enhancing its transcriptional activity.^[^ ^38^ ^]^ The high expression of osmotically responsive genes 1 (*HOS1*) encodes a RING finger‐type E3 ligase that ubiquitinates ICE1, leading to its cold‐induced degradation.^[^ ^39^ ^]^ However, phosphorylation by OST1 and sumoylation by the SUMO E3 ligase SIZ1 can inhibit this HOS1‐mediated ubiquitination and degradation of ICE1.^[^ ^2^, ^38^, ^40^ ^]^ Moreover, protein kinases MPK3/6 and BIN2 can phosphorylate ICE1, resulting in its degradation.^[^ ^41^, ^42^ ^]^ Besides mediating target protein degradation through the ubiquitination pathway, HOS1 can also reportedly affect the transcriptional activity of TFs through interaction. For example, HOS1 interacts with PIF4 to form complexes that inhibit PIF4's transcriptional activation activity, rather than regulating its protein turnover.^[^ ^43^, ^44^ ^]^ So far, no detailed functional studies of HOS1 have been conducted in apple, and it remains unclear whether HOS1‐mediated ubiquitination is involved in the regulation of HD‐Zip TFs.

Apples are one of the most economically significant fruit crops globally, and cold stress is a major environmental factor impacting their productivity and quality. For example, recent years have seen frequent early spring chilling and late spring frosts, which have significantly impeded the development of the apple industry in China.^[^ ^16^, ^19^, ^20^ ^]^ With advances in transgenic and gene editing technologies, their application in fruit crops like apples has facilitated the breeding of new varieties with enhanced resistance.^[^ ^45^, ^46^, ^47^ ^]^ This precise molecular breeding relies on identifying key genes involved in stress response and understanding their regulatory mechanisms. This study identifies MdHB7L, a HD‐Zip I TF, as a positive regulator of apple cold tolerance. MdHB7L interacts with MdICE1L and promotes the expression of MdCBF1/3 in both ICE1‐dependent and ICE1‐independent manners. Additionally, it enhances cold tolerance through a CBF‐independent pathway by stimulating antioxidant enzyme activity and the accumulation of proline, soluble sugars, and anthocyanins. We also identified MdHOS1 as a negative regulator of cold tolerance. MdHOS1 mediates the ubiquitination and degradation of both MdHB7L and MdICE1L, preferentially interacting with and promoting the degradation of MdICE1L. These findings, combined with the inhibitory effect of MdICE1L on MdHB7L's promoter‐binding ability, suggest that MdHB7L primarily acts as a TF after MdICE1L degradation during cold signaling, and as a cofactor of MdICE1L prior to that. Overall, this study offers a mechanistic understanding of how the MdHB7L–MdICE1–MdHOS1 module fine‐tunes the cold response via CBF‐dependent and CBF‐independent pathways.

## 2 Results

### 2.1 MdHB7L Positively Regulates Cold Tolerance in Apple

**Figure 1:**
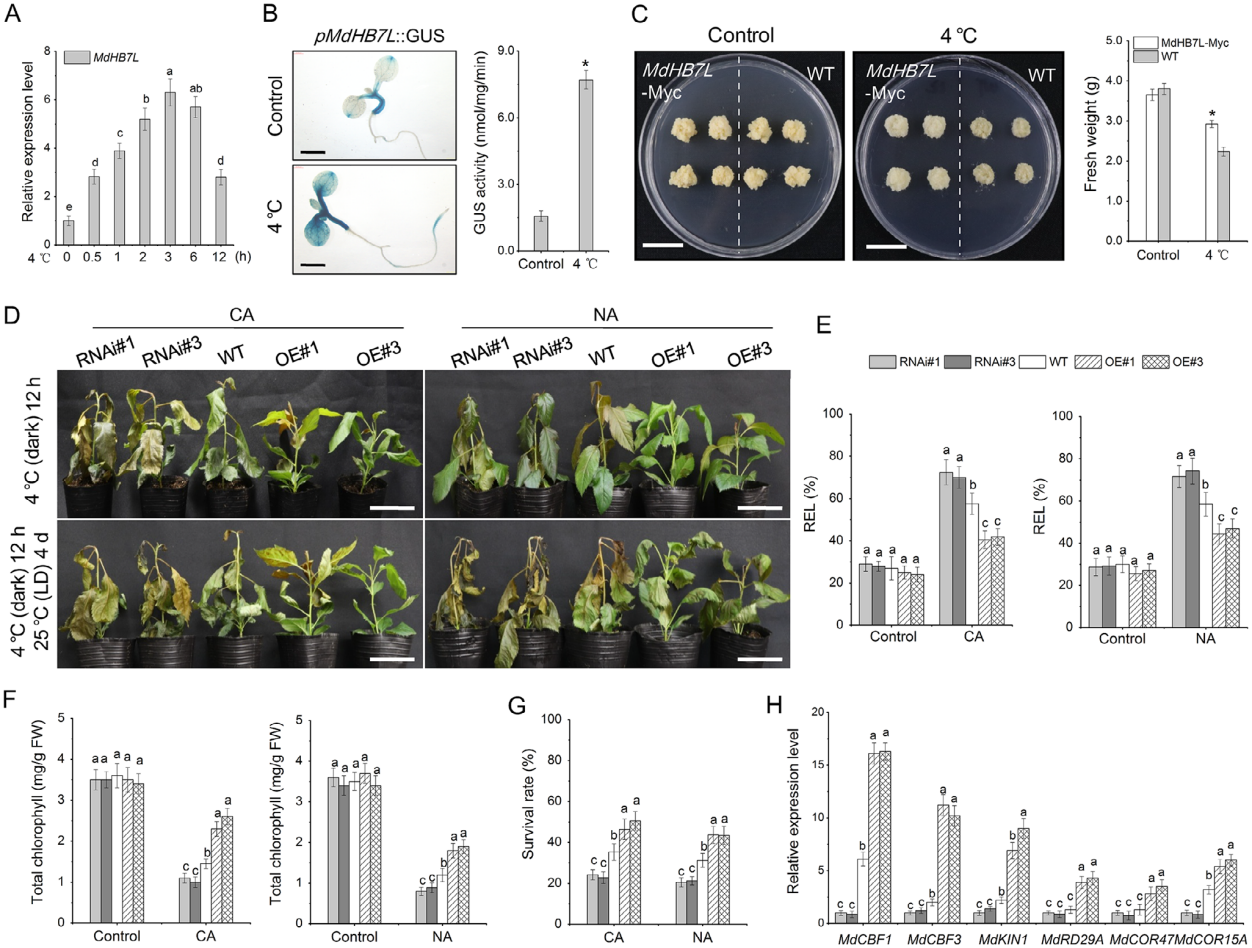
MdHB7L is a cold response gene that positively regulates apple cold tolerance. A) Analysis of MdHB7L expression in apple leaves subjected to 4 °C treatment. B) GUS staining analysis showing transcriptional activity of the MdHB7L promoter. scale bar: 2 mm. C) Growth phenotype of apple calli. Wild‐type (WT) and MdHB7L‐Myc transgenic calli were cultured under control (25 °C) and 4 °C conditions for 20 d. scale bar: 2 cm. D) Phenotypes of WT and MdHB7L transgenic apple plants after freezing treatment. Top images show plant phenotypes after a 12 h recovery under 4 °C in darkness after freezing treatment. Bottom images show phenotypes of plants cultured for four days under normal conditions following freezing treatment. CA, cold acclimation; NA, non‐cold acclimation. scale bar: 7 cm. E–G) Leaf relative electrolyte leakage (REL) (E), chlorophyll content (F), and survival rate (G) after freezing treatment. H) Expression analysis of MdCBFs and downstream COR genes after 6 h of 4 °C treatment. Error bars represent SD based on three biological replicates. Asterisks denote values significantly different from control (*p* < 0.05, Student's *t*‐test). Different letters indicate significant differences at *p* < 0.05, as determined by one‐way ANOVA and Duncan's tests.

Under 4 °C treatment, MdHB7L expression increased significantly and rapidly, peaking at 3 h and remaining elevated after 12 h of treatment (**Figure** **1**A). To further examine MdHB7L's response to cold stress, its promoter was cloned to drive *GUS* gene expression in *Arabidopsis* (Figure S1A, Supporting Information). Histochemical staining and *GUS* activity assays showed that cold treatment activated the MdHB7L promoter, primarily in leaves (Figure 1B).

To assess the role of MdHB7L in apple cold tolerance, wild‐type (WT), and MdHB7L‐Myc transgenic calli were exposed to cold stress. Treatment at 4 °C significantly inhibited calli growth, but this inhibition was less pronounced in MdHB7L‐Myc calli (Figure 1C), indicating that MdHB7L positively contributes to cold tolerance. Further investigation into MdHB7L's regulatory function in the apple cold response involved subjecting MdHB7L‐OE (overexpression) and MdHB7L‐RNAi (RNA interference) transgenic apple plants to freezing treatment, with (CA) and without (NA) prior cold acclimation. Phenotypic observations revealed that, compared to WT, MdHB7L‐RNAi plants exhibited more severe stress damage, while MdHB7L‐OE plants showed greater resilience (Figure 1D). Measurements of stress‐related physiological indexes supported these phenotypic differences: compared to WT, MdHB7L‐RNAi lines had higher relative electrolyte leakage (REL), lower chlorophyll content, and reduced survival rates, while MdHB7L‐OE lines displayed the opposite trends under both NA and CA treatment conditions (Figure 1E–G). We also measured the expression of several CBFs and downstream CORs under cold treatment. Their expression was significantly upregulated in MdHB7L‐OE plants and downregulated in MdHB7L‐RNAi plants compared to WT (Figure 1H). These results indicated that MdHB7L functions as a positive regulator in the apple cold response.

### 2.2 MdHB7L Interacts with MdICE1L to Promote MdICE1L‐Mediated Transcriptional Activation of MdCBFs

**Figure 2:**
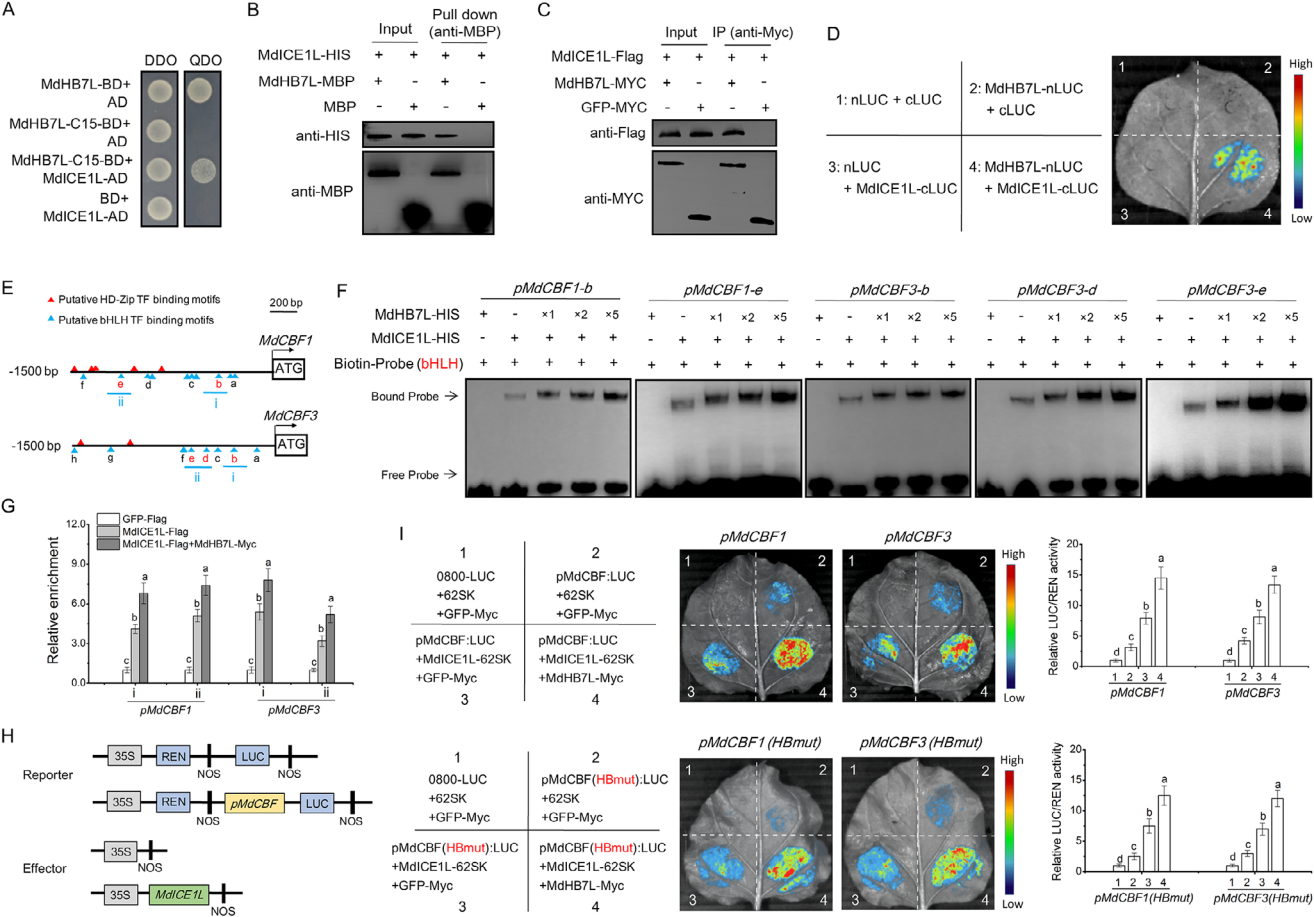
MdHB7L interacts with MdICE1L and promotes its binding and transcriptional activation activity on MdCBF1/3 promoters. A–D) Identification of protein interactions between MdHB7L and MdICE1L using Y2H (A), pull‐down (B), Co‐IP (C), and split‐LUC (D) assays. MdHB7L‐C15 refers to a truncated MdHB7L with 15 amino acids removed from the C‐terminus. AD and BD indicate the pGADT7 and pGBKT7 vectors, respectively. DDO represents SD medium lacking leucine and tryptophan, while QDO lacks leucine, tryptophan, histidine, and adenine. E) Diagram showing the putative bHLH and HD‐Zip TF binding sites in MdCBF1/3 promoters. The red letters mark the MdICE1L binding sites validated in a previous study.[^16^ ^]^ Regions i and ii denote the fragments amplified by ChIP‐qPCR in (G). F) EMSA results showing that MdHB7L enhances MdICE1L binding to the MdCBF1/3 promoters. × 1, × 2, and × 5 indicate increasing MdHB7L protein levels relative to MdICE1L. **(G)** ChIP‐qPCR assays demonstrating that MdHB7L enhances MdICE1L binding to MdCBF1/3 promoters in vivo. Apple calli transformed with GFP‐Flag served as a control, and relative enrichment was calculated as the ratio of MdICE1L/MdHB7L transgenic samples to controls. H) Schematic of reporter and effector vectors used in Dual‐LUC assays. I) Dual‐LUC assays indicating that MdHB7L promotes MdICE1L‐mediated transcriptional activation of MdCBF1/3 under cold stress. 0800‐LUC and 62SK are the empty reporter and effector vectors, respectively. “HBmut” denotes promoters with core nucleotides within putative HD‐Zip TF binding motifs replaced by C's. Data are presented as mean ± SD from three biological replicates. Different letters denote significant differences at *p* < 0.05 (Duncan's test).

To investigate the mechanism behind MdHB7L‐mediated cold tolerance, we used MdHB7L‐C15, a truncated MdHB7L variant without self‐activation activity (**Figure** **2**A),^[^ ^35^ ^]^ to screen the apple cDNA library via a Y2H system. MdICE1L, an AtICE1 ortholog in apple,^[^ ^16^ ^]^ was identified as an MdHB7L‐interacting protein. Subsequently, the interaction between MdHB7L and MdICE1L was confirmed in vitro through Y2H and pull‐down assays (Figure 2A,B). To confirm the interaction in vivo, we transiently expressed MdHB7L‐Myc and MdICE1L‐Flag vectors in tobacco leaves, detecting the MdHB7L–MdICE1L interaction through Co‐IP assays (Figure 2C). Moreover, this in vivo interaction was further validated by Split‐LUC assays (Figure 2D).

MdCBF1 and MdCBF3, previously identified as direct targets of MdICE1L, have two and three MdICE1L‐binding sites verified in their promoters, respectively (Figure 2E).^[^ ^16^ ^]^ We investigated how MdHB7L affects MdICE1L's binding ability to these promoter sites using EMSA assays. The MdHB7L–HIS protein alone could not bind to the bHLH‐specific probes (Figure 2F). However, adding MdHB7L–HIS significantly increased the band intensity of the MdICE1L‐probe complexes (Figure 2F), indicating that MdHB7L enhances MdICE1L binding activity through interaction. To confirm the effect of MdHB7L on MdICE1L's promoter binding activity in vivo, we performed ChIP‐qPCR assays using MdICE1L‐Flag and MdHB7L‐Myc + MdICE1L‐Flag transgenic calli (Figure S2A, Supporting Information). The results showed that MdHB7L co‐expression significantly promoted MdICE1L binding activity to MdCBF1/3 promoters (Figure 2G).

The role of MdHB7L in enhancing MdICE1L‐mediated transcriptional activation of MdCBFs was assessed using Dual‐LUC assays. Fluorescence observations and measurements of relative LUC/REN activity showed that MdICE1L increased transcriptional activity at the MdCBF1/3 promoters, which was further significantly enhanced by co‐expressing MdHB7L (Figure 2H,I). Given that the MdCBF1/3 promoters contain multiple *cis*‐elements that may be recognized by HD‐Zip TFs (Figure 2E), promoter sequences with mutated core nucleotides in these *cis*‐elements were synthesized to exclude the potential direct binding and regulation by MdHB7L. The results of Dual‐LUC assays showed that MdHB7L co‐expression still significantly enhanced the transcriptional activation effect of MdICE1L on the mutated MdCBF1/3 promoters (Figure 2I). These results indicated that MdHB7L interacts with MdICE1L to strengthen its binding and transcriptional activation at MdCBF1/3 promoters.

### 2.3 MdHB7L Activates MdCBFs Expression by Directly Binding to their Promoters, A Process that is Inhibited by MdICE1L

**Figure 3:**
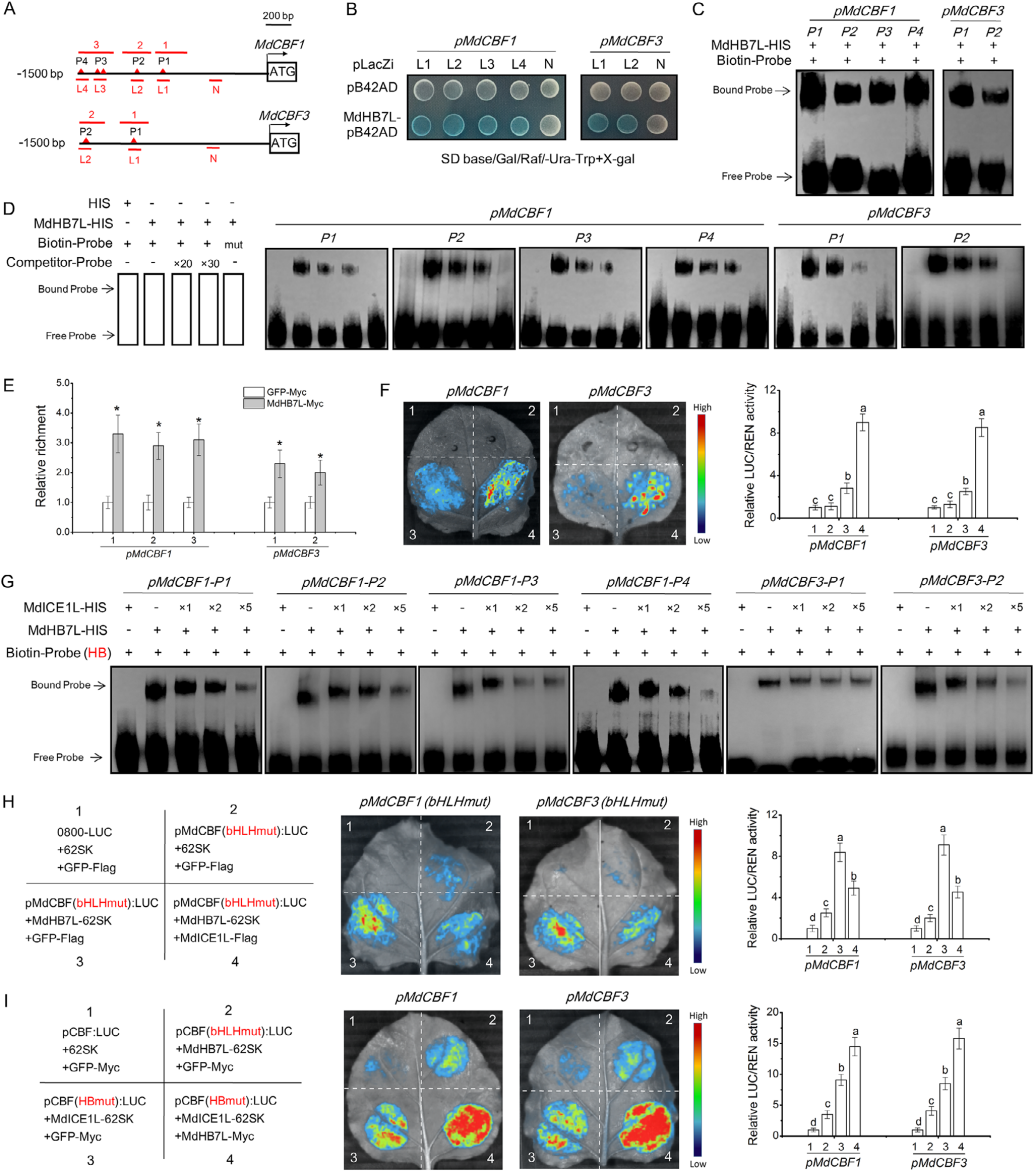
MdHB7L directly binds to the HD‐Zip TF binding motifs in MdCBF1/3 promoters to activate their transcription, a process that is inhibited by MdICE1L. A) Schematic of the putative HD‐Zip TF binding sites (P1 to P4) within the MdCBF1/3 promoters. L1 to L4 and N (control) indicate fragments cloned for the Y1H assays in (B). Red numbers denote fragments amplified by ChIP‐qPCR in (E). B,C) Y1H (B) and EMSA (C) assays demonstrating MdHB7L binding to the HD‐Zip TF binding motifs in MdCBF1/3 promoters. Fragments lacking HD‐Zip TF‐binding motifs were randomly chosen as controls and labeled “N”. D) Assessment of MdHB7L binding specificity through competitive EMSAs. HIS protein was used as a negative control. “Competitor” refers to probes without biotin labeling. × 20 and × 30 represent the ratio of competitors to normal probes. “Mut” represents a mutated probe. E) ChIP‐qPCR assays showing in vivo binding of MdHB7L to the MdCBF1/3 promoters. Apple calli transformed with GFP‐Myc served as the control. Error bars represent SD based on three biological replicates. Asterisks indicate values significantly different from the control (*p* < 0.05, Student's *t*‐test). F) Dual‐LUC assays showing that MdHB7L activates MdCBF1/3 promoter transcription under cold conditions. 1) empty reporter and effector vectors; 2) empty reporter + 35S::*MdHB7L*; 3) proMdCBF1/*3*::LUC + empty effector; 4) proMdCBF1/3::LUC + 35S::MdHB7L. G) EMSAs showing that MdICE1L inhibits MdHB7L binding to the HD‐Zip TF binding motifs in MdCBF1/3 promoters. H) Dual‐LUC assays showing that MdHB7L‐mediated transcriptional activation dependent on the HD‐Zip TF binding motifs was inhibited by MdICE1L. “bHLHmut” refers to promoters with core nucleotides in putative bHLH TF binding motifs replaced with A's, and “HBmut” denotes promoters with core nucleotides within putative HD‐Zip TF binding motifs replaced by C's. I) Dual‐LUC assays showing MdICE1L's stronger transcriptional activation activity compared to MdHB7L. In panels F, H, and I, data are presented as mean ± SD from three biological replicates. Different letters denote significant differences at *p* < 0.05 (Duncan's test).

In MdCBF1 and MdCBF3 promoters, four and two potential HD‐Zip TF binding sites (P1 to P4), respectively, were identified (**Figure** **3**A), raising the possibility of direct binding by MdHB7L. Several promoter fragments (L1 to L4) containing specific binding sites were cloned (Figure 3A). Y1H assay results confirmed that MdHB7L binds to each of these sites in MdCBF1/3 promoters (Figure 3B). Further validation of MdHB7L binding to these promoters was performed using EMSA assays, which demonstrated binding to the four and two sites in MdCBF1 and MdCBF3 promoters, respectively (Figure 3C). Binding specificity was confirmed through competitive EMSA assays with mutant and competitor probes (Figure 3D). Additionally, in vivo binding of MdHB7L to these sites was verified by ChIP‐qPCR assays using MdHB7L‐Myc and GFP‐Myc calli (Figure 3E; Figure S2B, Supporting Information). Finally, the transcriptional activation effect of MdHB7L on MdCBF1/3 promoters was evaluated with the Dual‐LUC system. The co‐expression of MdHB7L significantly enhanced the transcriptional activity of these promoters under cold conditions (Figure 3F). These results demonstrate that MdHB7L activated MdCBF1/3 expression by directly binding to their promoters.

Considering the MdHB7L–MdICE1L interaction, we investigated how MdICE1L influences MdHB7L's ability to bind to these HD‐Zip TF binding sites in MdCBF1/3 promoters. EMSA results revealed that MdICEL1 alone did not bind to the MdHB7L binding sites. Interestingly, the addition of MdICEL1 significantly reduced the band intensity of the MdHB7L‐probe complexes (Figure 3G), indicating that MdICE1L inhibited MdHB7L's binding capacity. To examine MdICE1L's effect on MdHB7L‐mediated transcriptional activation of MdCBFs dependent on the HD‐Zip TF binding motifs, promoter sequences with mutated core nucleotides in potential bHLH‐binding *cis*‐elements were synthesized to rule out direct binding and regulation by MdICE1L (Figure 2E). The results of Dual‐LUC assays showed that co‐expression with MdICE1L significantly inhibited MdHB7L‐mediated transcriptional activation of the mutated MdCBF1/3 promoters (Figure 3H). Furthermore, Dual‐LUC assays using the synthesized promoters demonstrated that MdICE1L's transcriptional activation of MdCBF1/3 promoters was significantly higher than that of MdHB7L, especially when MdHB7L was co‐expressed (Figure 3I). These findings suggest that the MdHB7L–MdICE1L complex tends to bind to the MdICE1L‐binding sites, but not the MdHB7L‐binding sites, to activate MdCBFs experssion. This also indicates that MdHB7L acts as a cofactor rather than a transcription factor when interacting with MdICE1L.

### 2.4 ChIP‐seq and RNA‐seq Demonstrate that MdHB7L Promotes Antioxidant Enzyme Activity by Directly Activating *MdPRX52/53* and *MdRD26* Expression

**Figure 4:**
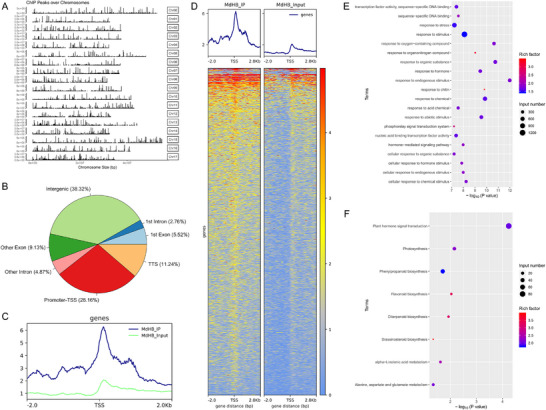
ChIP‐seq identified many potential target genes of MdHB7L in apple at the genome‐wide level. A) Diagram showing the distribution of identified peaks across different apple chromosomes. Apple has 17 chromosomes (Chr01–Chr17), with Chr00 representing the unassembled genomic scaffold. B) Statistical analysis of identified peak distribution across various functional regions of the genome. TTS, transcription termination site. C) Distribution of reads around the transcription start site (TSS). The green and blue lines indicate coverage depth of reads in input and IP samples, respectively. D) Diagram depicting read distribution upstream and downstream of the TSS for each gene. E,F) GO (E) and KEGG pathway (F) enrichment analyses of the 4219 genes associated with the identified peaks.

To investigate the mechanism underlying MdHB7L‐mediated cold tolerance, we performed ChIP‐seq to identify potential MdHB7L target genes across the genome. A total of 9035 peaks were identified using default screening parameters (Fold enrichment (FE) > 1.5, *p* < 0.05), which were widely distributed across 17 chromosomes of apple (**Figure** **4**A; Table S1, Supporting Information). Most peaks were located in intergenic (38.32%) and promoter (28.16%) regions (Figure 4B). Moreover, the reads distribution analysis revealed significant enrichment of MdHB7L‐bound DNA sequences near the transcription start site (TSS) (Figure 4C,D). We identified 4219 genes associated with these peaks, including *MdCBF1* (*MD07G1262900*) (Table S1, Supporting Information), confirming the reliability of our ChIP‐seq results. Gene Ontology (GO) enrichment analysis showed that these genes were primarily involved in responses to various stresses and hormones (Figure 4E; Table S2, Supporting Information). Additionally, KEGG pathway enrichment analysis indicated that most genes were involved in hormone biosynthesis and signaling, and the biosynthesis and metabolism of phenylpropanoid, flavonoids, diterpenoids, α‐Linolenic acid, and various amino acids (Figure 4F; Table S3, Supporting Information), all crucial for cold tolerance.

**Figure 5:**
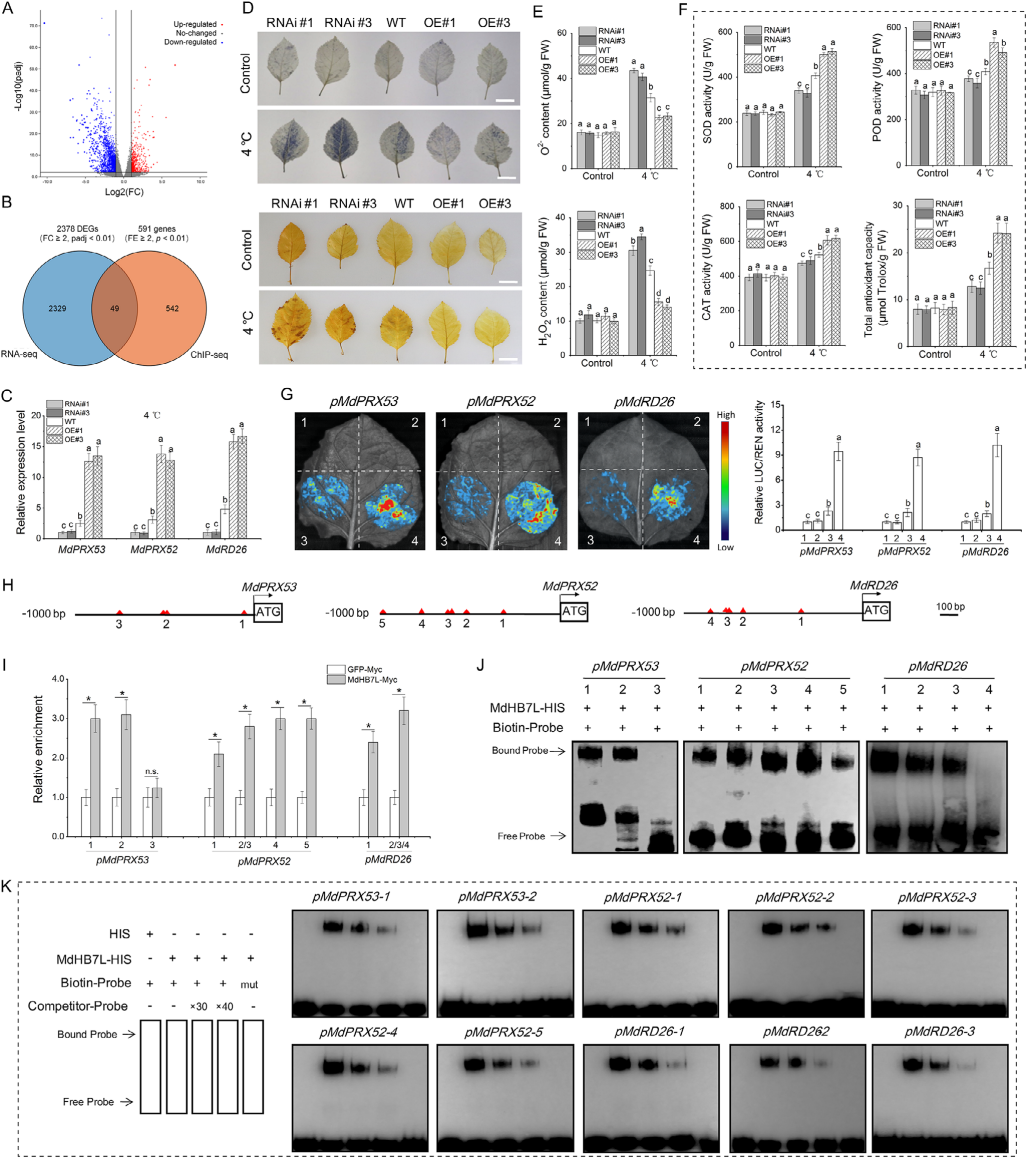
MdHB7L promotes antioxidant enzyme activity by directly activating ***MdPRX52/53*** and ***MdRD26*** expression. A) Volcano plot of DEGs identified with screening thresholds of fold change (FC) ≥ 2 and adjusted *p*‐value (padj) < 0.01. B) Venn diagrams showing genes identified by ChIP‐seq and RNA‐seq screening. DEGs, differentially expressed genes. C) Expression analysis of *MdPRX52/53* and *MdRD26* under 4 °C treatment for 6 h. D) In situ detection of O_2_^−^ and H_2_O_2_ accumulation by NBT (top) and DAB (bottom) histochemical staining, respectively. scale bar: 2 cm. E) Quantification of O_2_^−^ and H_2_O_2_ content in apple leaves. F) Enzyme activity assays for superoxide dismutase (SOD), peroxidase (POD), and catalase (CAT), along with total antioxidant capacity in apple leaves. In panels D to F, leaves from apple plants treated at 4 °C for 24 h were used. G) Dual‐LUC assays demonstrating that MdHB7L enhances transcription of the *MdPRX52/53* and *MdRD26* promoters under cold conditions. 1) empty reporter and effector vectors; 2) empty reporter + 35S::MdHB7L; 3) *pro*::LUC + empty effector; 4) pro::LUC + 35S::MdHB7L, where “pro” represents the promoters of *MdPRX52/53* or *MdRD26*. H) Diagram of putative HD‐Zip TF binding sites in the *MdPRX52/53* and *MdRD26* promoters. I) ChIP‐qPCR assays showing MdHB7L binding to the *MdPRX52/53* and *MdRD26* promoters in vivo. Numbers indicate potential binding sites as shown in (H). J) EMSAs showing MdHB7L binding to the *MdPRX52/53* and *MdRD26* promoters. K) Identification of the binding specificity of MdHB7L through competing EMSAs. Error bars represent SD based on three biological replicates. Different letters indicate significant differences at *p* < 0.05, as determined by one‐way ANOVA and Duncan's tests. Asterisks denote values significantly different from control (*p* < 0.05, Student's *t*‐test).

To further investigate potential target genes regulated by MdHB7L, we analyzed transcriptome data from MdHB7L‐OE and WT plants obtained in a previous study,^[^ ^36^ ^]^ in conjunction with ChIP‐seq data. Using stringent screening criteria (RNA‐seq: Fold Change (FC) ≥ 2, padj < 0.01; ChIP‐seq: FE ≥ 2, *p* < 0.01, promoter region), we identified 2378 differentially expressed genes (DEGs) from RNA‐seq data and 591 potential MdHB7L‐target genes from ChIP‐seq data (**Figure** **5**A; Tables S4,S5, Supporting Information). Venn analysis revealed 49 common genes, including two peroxidase‐encoding genes, MdPRX52 (MD04G1170900) and MdPRX53 (MD03G1013600), and a typical stress‐response gene, *MdRD26* (MD15G1079400), all of which were upregulated in MdHB7L‐OE plants (Figure 5B; Table S6, Supporting Information). RT‐qPCR analysis confirmed that the expression of these genes was significantly higher in MdHB7L‐OE plants and lower in MdHB7L‐RNAi plants compared to WT under cold conditions (Figure 5C). Additionally, DAB and NBT histochemical staining and measurements of H_2_O_2_ and O_2_^−^ contents showed significantly reduced excessive ROS accumulation in MdHB7L‐OE plants under cold stress (Figure 5D,E). Furthermore, the activity of antioxidant enzymes SOD, POD, and CAT, as well as the total antioxidant capacity, were significantly higher in MdHB7L‐OE plants compared to WT (Figure 5F). Conversely, these stress‐related indices showed the opposite trend in MdHB7L‐RNAi plants (Figure 5D–F).

To investigate the transcriptional regulation of MdHB7L on *MdPRX52*/*53* and *MdRD26*, we cloned their promoters and conducted Dual‐LUC assays. The results demonstrated that co‐expression with MdHB7L significantly enhanced the transcriptional activity of these promoters (Figure 5G). Upon analyzing the sequences, we identified several potential MdHB7L‐binding sites within these promoters (Figure 5H). Therefore, ChIP‐qPCR assays were carried out, and the results showed that MdHB7L directly binds to these promoters in vivo (Figure 5I). Additionally, EMSA assays demonstrated that MdHB7L binds to most of these sites in vitro, except for site 3 in the MdPRX53 promoter and site 4 in the *MdRD26* promoter (Figure 5J). Competitive EMSAs further verified the specificity of these binding interactions (Figure 5K). Therefore, these results suggest that MdHB7L promotes the scavenging of excessive ROS under cold stress by directly upregulating the expression of *MdPRX52*/*53* and *MdRD26*.

### 2.5 MdHB7L Promotes the Accumulation of Anthocyanins, Soluble Sugars, and Proline by Regulating the Expression of Genes Related to their Biosynthesis and Metabolism

**Figure 6:**
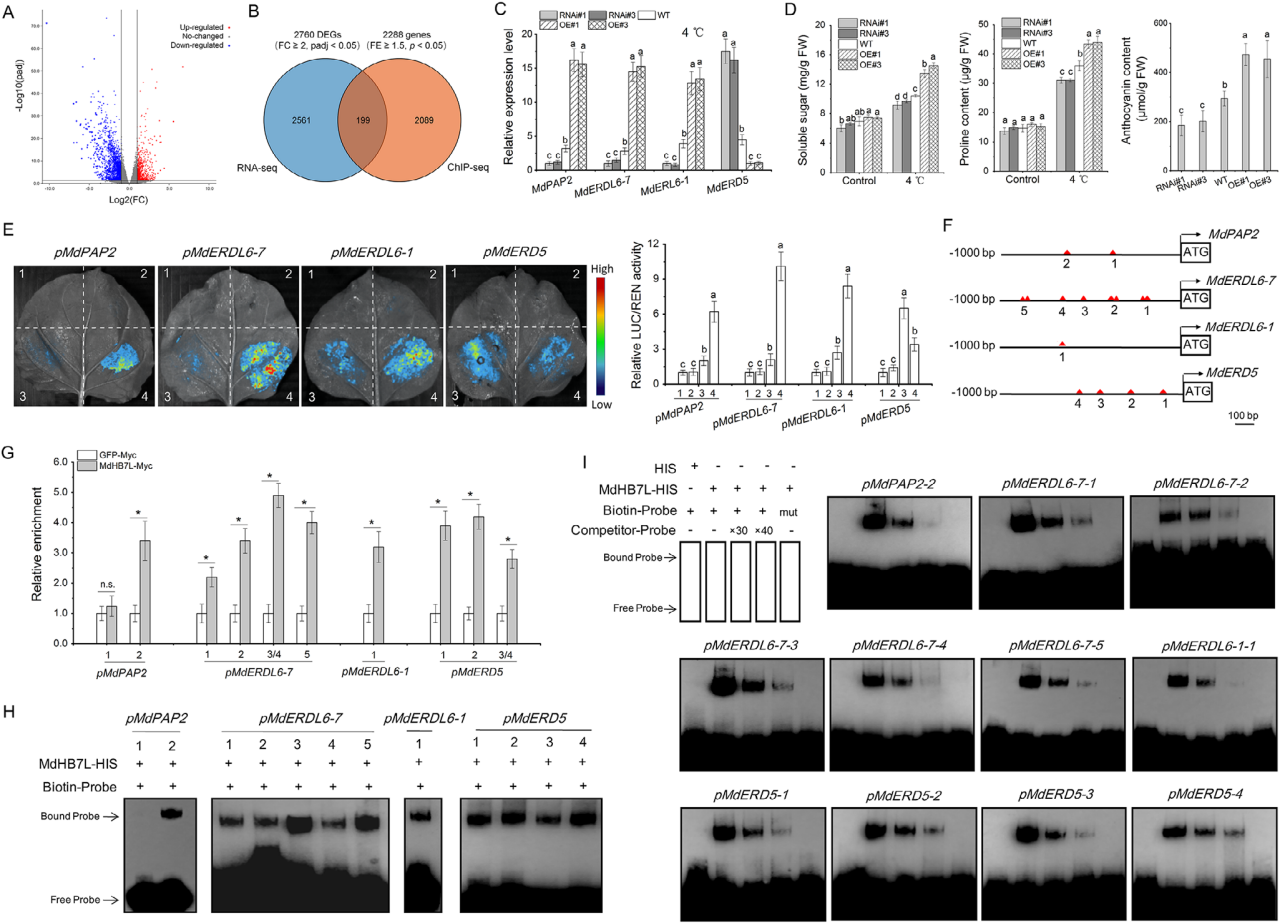
MdHB7L promotes the accumulation of anthocyanins, soluble sugars, and proline by regulating the expression of MdPAP2, MdERD6‐1/‐7, and *MdERD5*. A) Volcano plot of DEGs identified using a screening threshold of FC ≥ 2 and padj < 0.05. B) Venn diagram showing overlap of genes identified in ChIP‐seq and RNA‐seq analyses. C) Expression analysis of MdPAP2, MdERD6‐1/‐7, and *MdERD5* under 4 °C treatment for 6 h. D) Measurement of soluble sugar, proline, and anthocyanin contents in apple leaves. Plants treated at 4 °C for 35 d under LD conditions were used. E) Dual‐LUC assays demonstrating the effect of MdHB7L on the transcriptional activity of MdPAP2, MdERD6‐1/‐7, and *MdERD5* promoters under cold conditions. 1) empty reporter and effector vectors; 2) empty reporter + 35S::MdHB7L; 3) pro::LUC + empty effector; 4) pro::LUC + 35S::MdHB7L. “pro” refers to the promoters of MdPAP2, MdERD6‐1/‐7, or *MdERD5*. F) Diagram depicting the distribution of putative HD‐Zip TF binding sites. G) ChIP‐qPCR assays showing in vivo binding of MdHB7L to the promoters of MdPAP2, MdERD6‐1/‐7, and *MdERD5*. Numbers indicate potential binding sites as shown in (F). H) EMSAs demonstrating the binding of MdHB7L to the promoters of MdPAP2, MdERD6‐1/‐7, and *MdERD5*. I) Competitive EMSAs identifying the binding specificity of MdHB7L. Error bars represent SD based on three biological replicates. Different letters indicate significant differences at *p* < 0.05, as determined by one‐way ANOVA and Duncan's tests. Asterisks denote values significantly different from control (*p* < 0.05, Student's *t*‐test).

To further investigate how MdHB7L enhances cold tolerance, a lower screening threshold was applied (RNA‐seq: FC ≥ 2, padj < 0.05; ChIP‐seq: FE ≥ 1.5, *p* < 0.05, promoter region), identifying 2760 DEGs and 2288 potential MdHB7L‐target genes from RNA‐seq and ChIP‐seq data, respectively (**Figure** **6**A; Tables S7,S8, Supporting Information). Venn analysis revealed 199 genes common to both datasets (Figure 6B; Table S9, Supporting Information). Notably, MdPAP2 (MD17G1261000) and MdERDL6‐7 (MD13G1175900), which promote anthocyanin and soluble sugar accumulation respectively,^[^ ^48^, ^49^, ^50^ ^]^ were upregulated in MdHB7L‐OE plants. Conversely, *MdERD5* (MD17G1070700), a gene encoding proline dehydrogenase involved in proline metabolism,^[^ ^51^, ^52^ ^]^ was downregulated (Table S9, Supporting Information). Additionally, another ERDL family gene, MdERDL6‐1 (MD15G1026400), which promotes soluble sugar accumulation in apples,^[^ ^50^ ^]^ was detected in the ChIP‐seq results (Table S8, Supporting Information). RT‐qPCR analysis confirmed that, compared to WT, MdPAP2 and MdERDL6‐1/6‐7 expression was significantly higher in MdHB7L‐OE plants but lower in MdHB7L‐RNAi plants under cold conditions. Conversely, *MdERD5* expression showed the opposite trend (Figure 6C). Consistent with these gene expression patterns, anthocyanin, soluble sugar, and proline accumulation significantly increased in MdHB7L‐OE plants and decreased in MdHB7L‐RNAi plants under cold stress (Figure 6D).

To determine the regulatory role of MdHB7L on specific genes, their promoters were cloned. Dual‐LUC assays revealed that MdHB7L co‐expression enhanced the transcriptional activity of MdPAP2, MdERDL6‐1, and MdERDL6‐7 promoters but inhibited the *MdERD5* promoter (Figure 6E). Multiple potential MdHB7L‐binding sites were identified in these promoters (Figure 6F). ChIP‐qPCR assays confirmed that MdHB7L directly binds to most of these sites in vivo (Figure 6G). Additionally, EMSA results showed that MdHB7L directly binds to these sites, except for site 1 in the MdPAP2 promoter (Figure 6H), with binding specificity verified by competing EMSAs (Figure 6I). These results suggest that MdHB7L promotes the accumulation of anthocyanins, soluble sugars, and proline under cold conditions by directly regulating the expression of MdPAP2, MdERDL6‐1/6‐7, and *MdERD5*.

### 2.6 MdHB7L Interacts with MdHOS1 and is Competitively Inhibited by MdICE1L

**Figure 7:**
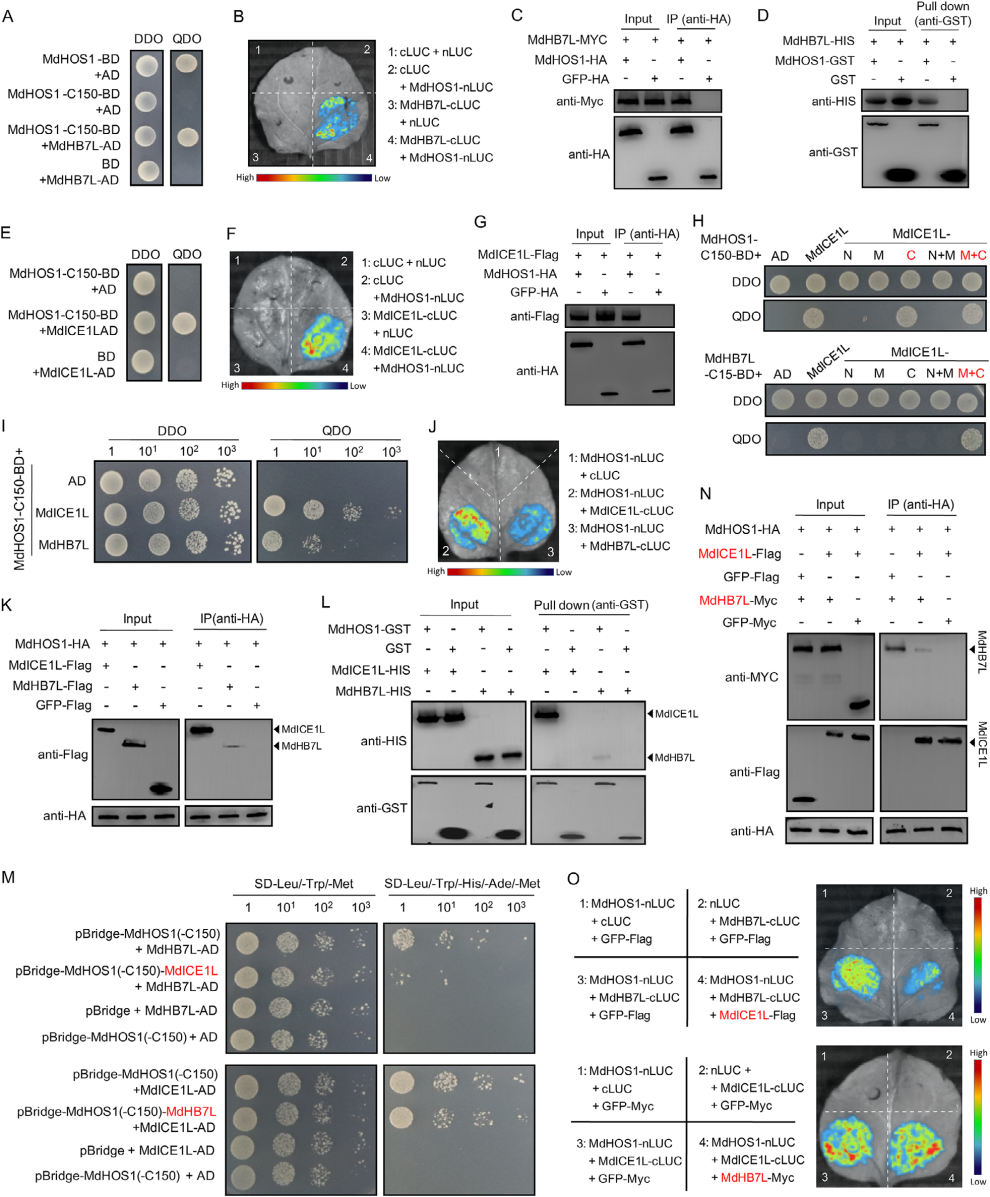
MdHB7L interacts with MdHOS1 and is competitively inhibited by MdICE1L. A–D) Identification of protein interactions between MdHB7L and MdHOS1 using Y2H (A), split‐LUC (B), Co‐IP (C), and pull‐down (D) assays. MdHOS1‐C150 is a truncated form of MdHOS1 with 150 amino acids removed from the C‐terminal region. E–G) Identification of protein interactions between MdICE1L and MdHOS1 using Y2H (E), split‐LUC (F), and Co‐IP (G) assays. H) Identification of critical regions in MdICE1L responsible for interacting with MdHOS1 and MdHB7L through Y2H assays. The N, M, and C labels refer to the N‐terminal, middle, and C‐terminal regions of MdICE1L, respectively. I–L) Comparison of interaction activity between MdHOS1–MdICE1L and MdHOS1–MdHB7L using Y2H (I), split‐LUC (J), Co‐IP (K), and pull‐down (L) assays. M–O) Identification of the inhibitory effect of MdICE1L on the MdHOS1–MdHB7L interaction using Y3H (M), Co‐IP (N), and split‐LUC (O) assays.

In addition to MdICE1L, the E3 ligase MdHOS1 (MD04G1060900) was identified as an MdHB7L‐interacting protein via Y2H screening. MdHOS1 was cloned from apple, and its direct interaction with MdHB7L was verified both in vitro and in vivo through Y2H, Split‐LUC, Co‐IP, and Pull‐down assays (**Figure** **7**A–D). Given the known ICE1‐HOS1 interaction in *Arabidopsis*, the interaction between MdICE1L and MdHOS1 was also tested. As anticipated, Y2H, Split‐LUC, and Co‐IP assays confirmed that MdICE1L interacts with MdHOS1 (Figure 7E–G).

The interactions observed between MdHB7L, MdICE1L, and MdHOS1 proteins suggest a mutual influence among them. To identify crucial interaction regions, truncated fragments of MdICE1L, obtained from a previous study,^[^ ^16^ ^]^ were used in Y2H assays. The C‐terminal region of MdICE1L was found to be key for both MdHB7L–MdICE1L and MdHOS1–MdICE1L interactions (Figure 7H), indicating potential antagonism between these interactions. Comparing the interaction activity of MdHB7L and MdICE1L with MdHOS1, we found that MdHOS1 interacts more strongly with MdICE1L than with MdHB7L. This was consistently demonstrated by Y2H, Split‐LUC, Co‐IP, and Pull‐down assays (Figure 7I–L). Moreover, Y3H assays revealed that co‐expressing MdICE1L significantly inhibited the MdHOS1–MdHB7L interaction. Conversely, co‐expressing *MdHB7L* had little impact on the interaction between MdICE1L and MdHOS1 (Figure 7M). To confirm these findings in vivo, Co‐IP and Split‐LUC assays were conducted. Protein band intensity detection and fluorescence observation further supported the earlier results: MdICE1L significantly inhibited the MdHOS1–MdHB7L interaction, while MdHB7L had minimal effect on the MdHOS1–MdICE1L interaction (Figure 7N–O). These results collectively indicate that MdHOS1 preferentially interacts with MdICE1L, thereby inhibiting the interaction of MdHB7L with MdHOS1.

### 2.7 Cold Treatment Inhibits MdHOS1‐Meidated Ubiquitination and Degradation of MdHB7L

**Figure 8:**
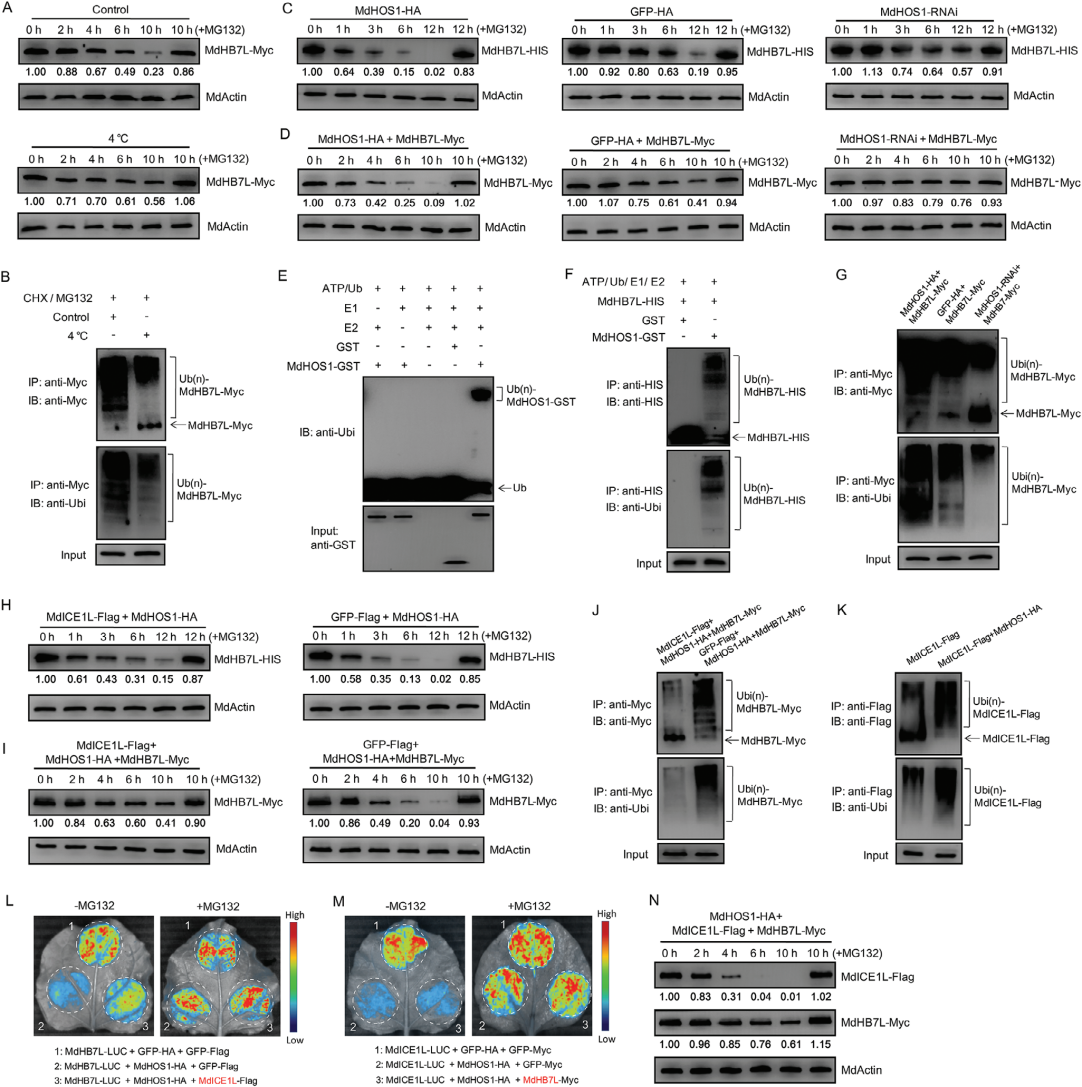
Cold and MdICE1L inhibit MdHOS1‐mediated protein degradation and ubiquitination of MdHB7L. A,B) Cold treatment inhibited the degradation (A) and ubiquitination (B) of MdHB7L. The protein levels of MdHB7L‐Myc in transgenic calli were measured at specified time points under 4 °C or normal conditions (+ 250 µm CHX). C) Cell‐free degradation assays of MdHB7L–HIS protein. Protein extracts from MdHOS1‐HA/RNAi and GFP–HA transgenic calli were incubated with MdHB7L–HIS protein for the indicated time periods at 22 °C. D) MdHOS1 promotes MdHB7L protein degradation in vivo. Protein levels of MdHB7L‐Myc were measured at specified time points under 4 °C treatment (+ 250 µm CHX). E,F) In vitro ubiquitination assays showing that MdHOS1 mediates the ubiquitination of both itself (E) and MdHB7L (F). G) In vivo ubiquitination assays showing that MdHOS1 promotes MdHB7L ubiquitination. Apple calli treated with CHX plus MG132 for 8 h under 4 °C treatment were collected for immunoprecipitation and ubiquitination detection. H,I) Cell‐free (H) and in vivo (I) degradation assays showing that MdICE1L inhibits MdHOS1‐mediated degradation of MdHB7L. J) MdICE1L inhibits MdHOS1‐mediated ubiquitination of MdHB7L in vivo. K) MdHOS1 promotes MdICE1L ubiquitination in vivo. Apple calli treated with CHX plus MG132 for 4 h under 4 °C treatment were used. L,M) Fluorescence observation of MdHB7L‐LUC (L) and MdICE1L‐LUC (M) in N. benthamiana leaves. N) Determination of protein levels of MdICE1L and MdHB7L in calli under cold conditions. In panels A, C, D, H, I, and N, numbers under the protein bands represent the band intensity, as determined by the ImageJ software.

To investigate the influence of cold on MdHB7L stability, MdHB7L‐Myc transgenic calli was used. Compared to the control, treatment at 4 °C significantly reduced the degradation rate of MdHB7L protein (**Figure** **8**A). The addition of MG132, a 26S proteasome inhibitor, inhibited and even blocked MdHB7L degradation. This suggests that MdHB7L was degraded through the ubiquitin‐26S proteasome pathway (Figure 8A). Correspondingly, detection of ubiquitination in calli showed that 4 °C treatment notably inhibited the ubiquitination level of MdHB7L (Figure 8B).

Since HOS1 is an E3 ubiquitin ligase, we examined its role in the ubiquitination and degradation of MdHB7L. First, a cell‐free method was used to assess the effect of MdHOS1 on MdHB7L protein stability. Purified MdHB7L–HIS was incubated with total proteins extracted from control (GFP‐HA), MdHOS1‐HA, and MdHOS1‐RNAi calli, respectively (Figure S2C, Supporting Information). Western‐blot results showed that overexpressing MdHOS1 promoted, while interfering with MdHOS1 expression inhibited, MdHB7L degradation (Figure 8C). The negative role of MdHOS1 on MdHB7L stability was also confirmed in vivo using MdHB7L‐Myc + MdHOS1(‐HA/‐RNAi) double transgenic calli (Figure 8D; Figure S2C, Supporting Information). The addition of MG132 eliminated the promotional effect of MdHOS1 on MdHB7L degradation (Figure 8C,D). This indicates that the 26S proteasome pathway is involved in MdHOS1‐mediated degradation of MdHB7L. In vitro ubiquitination assays showed that MdHOS1 could mediate self‐ubiquitination, indicating that MdHOS1 has E3 ubiquitin ligase activity (Figure 8E). Additionally, clear bands of poly‐ubiquitinated MdHB7L proteins were detected in the in vitro ubiquitination assays with the addition of MdHOS1–GST (Figure 8F). This indicates that MdHOS1 directly mediates MdHB7L ubiquitination. Furthermore, determination of ubiquitination in MdHB7L‐Myc + MdHOS1(‐HA/‐RNAi) transgenic calli showed that MdHOS1 promoted the ubiquitination of MdHB7L in vivo (Figure 8G). These results indicated that MdHOS1 mediated the ubiquitination and degradation of MdHB7L, a process that can be inhibited by cold.

### 2.8 MdICE1L Inhibits MdHOS1‐Meidated Ubiquitination and Degradation of MdHB7L

Given that MdICE1L inhibits the MdHOS1–MdHB7L interaction, we investigated its role in MdHOS1‐mediated MdHB7L degradation. Total proteins were extracted from MdICE1L‐Flag + MdHOS1‐HA and GFP‐Flag + MdHOS1‐HA (control) calli (Figure S2D, Supporting Information) and incubated with purified MdHB7L–HIS protein under cell‐free conditions, respectively. Measuring MdHB7L–HIS protein levels revealed that overexpressing MdICE1L significantly enhanced MdHB7L stability (Figure 8H). To confirm this finding in vivo, we transiently expressed MdICE1L‐Flag in MdHB7L‐Myc + MdHOS1‐HA transgenic calli using A. tumefaciens‐mediated transformation method (Figure S2D, Supporting Information). Compared to calli transiently transformed with GFP‐Flag, the degradation of MdHB7L was significantly reduced in triple transgenic calli co‐expressing MdICE1L (Figure 8I). Additionally, ubiquitination assays showed that co‐expressing MdICE1L in calli substantially inhibited MdHOS1‐mediated ubiquitination of MdHB7L (Figure 8J). Furthermore, we examined the role of MdHOS1 in mediating ubiquitination of MdICE1L in calli and found that MdHOS1 notably promoted MdICE1L ubiquitination (Figure 8K).

To further investigate the inhibitory role of MdICE1L in MdHOS1‐mediated MdHB7L degradation, a modified LUC assay was employed.^[^ ^53^ ^]^ The MdHB7L‐LUC expression vector was co‐transformed with MdHOS1‐HA and MdICE1L‐Flag vectors into N. benthamiana leaves in specific combinations for transient expression. Fluorescence observations revealed that MdHOS1 inhibited MdHB7L‐LUC accumulation, and this inhibition was significantly reduced by the co‐expression of MdICE1L (Figure 8L). To determine if MdHB7L affects MdICE1L stability, the same method was used. MdICE1L‐LUC accumulation was significantly inhibited by MdHOS1 co‐expression, and this inhibition was eliminated by MG132 treatment (Figure 8M), indicating that MdHOS1 mediates MdICE1L degradation via the 26S proteasome pathway. Co‐expression of MdHB7L did not alter the inhibitory effect of MdHOS1 on MdICE1L‐LUC accumulation (Figure 8M), suggesting that MdHOS1 preferentially mediates MdICE1L degradation over MdHB7L. We compared the degradation of MdHB7L and MdICE1L in the MdHB7L‐Myc + MdICE1L‐Flag calli transiently transformed with MdHOS1‐Flag (Figure S2E, Supporting Information). As expected, MdICE1L protein degradation occurred earlier and more rapidly than that of MdHB7L under cold conditions (Figure 8N). These results collectively suggest that when MdICE1L was abundant in early cold response, MdHOS1 preferentially interacts with and mediates the degradation of MdICE1L, thereby inhibiting MdHOS1‐mediated MdHB7L degradation.

### 2.9 MdHOS1 Attenuates MdHB7L‐Meidated Cold Tolerance and Transcriptional Activation of Target Genes

To determine the role of MdHOS1 in cold response, several transgenic *Arabidopsis* lines were generated (Figure S1B, Supporting Information). Three lines with high MdHOS1 expression were chosen for freezing treatment under both CA and NA conditions (Figures S1C,S3, Supporting Information). Under normal conditions, there was no significant difference between WT and transgenic seedlings. However, after freezing treatment, MdHOS1‐overexpressing *Arabidopsis* showed reduced cold tolerance, as indicated by phenotypic observations and measurements of survival rate and leaf REL (Figure S3A,B, Supporting Information). To investigate MdHOS1's function in apple, MdHOS1‐OE/RNAi transgenic calli were exposed to cold stress. At 4 °C, the growth of MdHOS1‐OE calli was more severely inhibited than WT, while MdHOS1‐RNAi calli were less affected (Figure S3C,D, Supporting Information). Furthermore, the MDA content in MdHOS1‐OE and MdHOS1‐RNAi calli were significantly higher and lower than that in WT calli, respectively (Figure S3E, Supporting Information).

**Figure 9:**
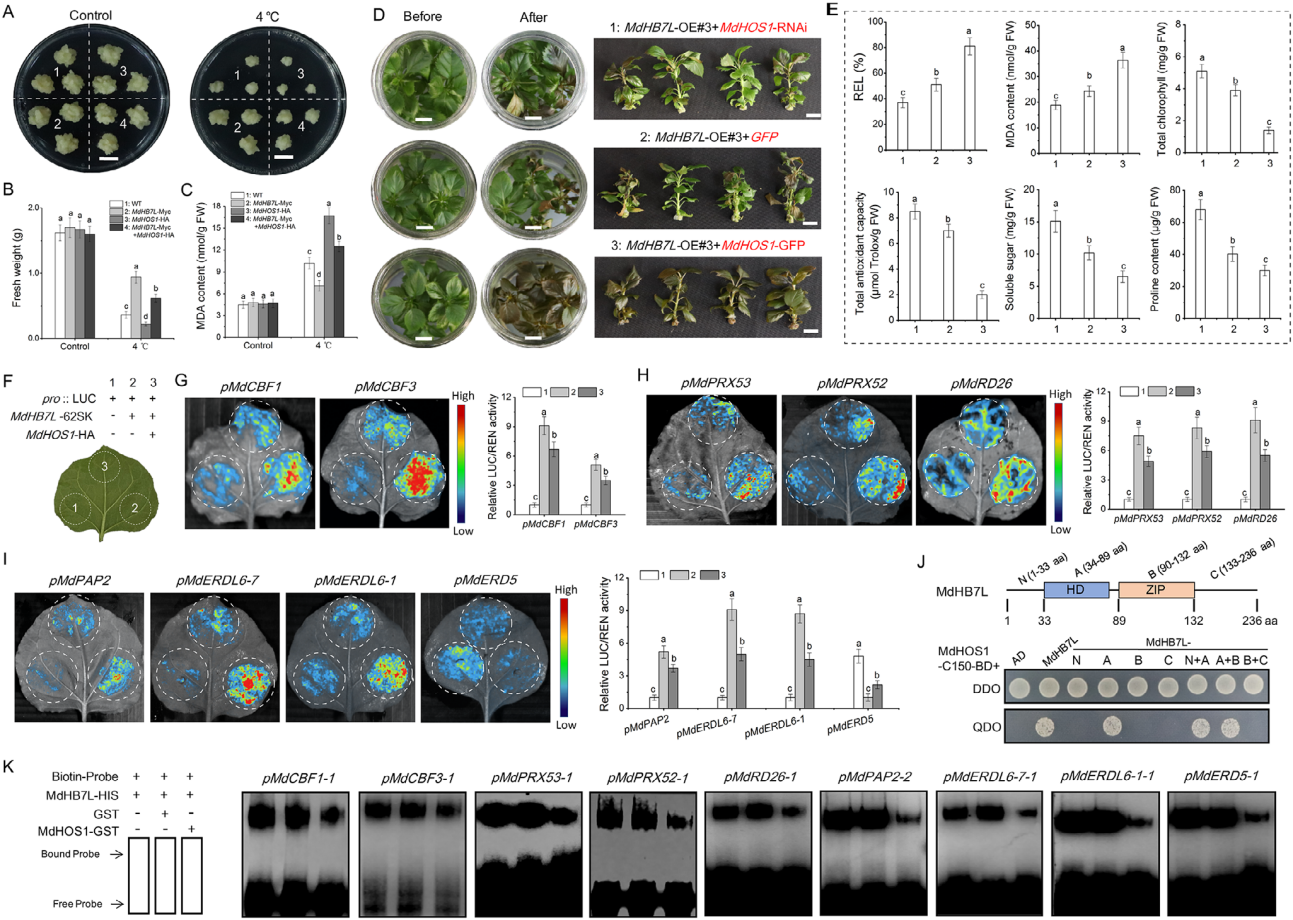
MdHOS1 attenuates the effects of MdHB7L on apple cold tolerance and transcriptional regulation of downstream target genes. A–C) Growth phenotypes (A), fresh weight (B), and malondialdehyde (MDA) content (C) of calli. scale bar = 1 cm. 1) WT; 2) MdHB7L‐Myc; 3) MdHOS1‐HA; 4) MdHB7L‐Myc + MdHOS1‐HA. D) The phenotypes of MdHB7L and MdHOS1 transgenic apple plants following freezing treatment. scale bars = 1 cm. E) Measurement of cold stress‐related physiological indices. F) Schematic of different vector combinations used for transient expression in Dual‐LUC assays in panels G to I. G–I) Fluorescence observation and determination of relative LUC/REN activity. In panels A to I, error bars represent SD based on three biological replicates. Different letters indicate significant differences at *p* < 0.05, as determined by one‐way ANOVA and Duncan's tests. J) Y2H assays demonstrating that the HD domain of MdHB7L is crucial for the MdHB7L–MdHOS1 interaction. K) EMSAs showing that MdHOS1 inhibits the binding of MdHB7L to the promoters of downstream target genes.

To explore MdHOS1's influence on MdHB7L‐mediated cold tolerance, various transgenic calli were exposed to cold stress. Compared to MdHB7L‐OE calli, overexpression of MdHOS1 in MdHB7L‐OE calli exhibited lower fresh weight and higher MDA content (**Figure** **9**A–C), suggesting that MdHOS1 weakens MdHB7L‐enhanced cold tolerance in apple. To confirm this in apple plants, MdHOS1‐GFP/RNAi vectors were constructed and transformed into MdHB7L‐OE plants using a vacuum‐based method (Figure S4, Supporting Information). Post‐freezing treatment, phenotypic observations and measurements of REL, MDA, and chlorophyll content indicated that co‐expressing MdHOS1 reduced MdHB7L‐promoted cold tolerance (Figure 9D,E). Furthermore, MdHOS1 co‐expression inhibited MdHB7L's positive effects on total antioxidant capacity and the accumulation of soluble sugars and proline under cold stress, while interfering with MdHOS1 expression reversed these trends (Figure 9D,E). To further investigate the relationship between MdHB7L, MdICE1L, and MdHOS1 in apple cold stress response, various transgenic lines with expression of these genes interfered were created by transient transformation (Figure S5A, Supporting Information), and leaf REL assays were performed. After freezing treatment, the REL of MdHB7L‐RNAi, MdICE1L‐RNAi, and MdHB7L‐RNAi + MdICE1L‐RNAi double transgenic lines was significantly increased compared with WT, while that of MdHOS1‐RNAi plants was significantly decreased. The REL of triple transgenic leaves showed the same trend as that of the double transgenic line (Figure S5B, Supporting Information), indicating that the regulatory role of MdHOS1 in apple cold response depends on MdHB7L and MdICE1L.

Dual‐LUC assays were conducted to examine the effect MdHOS1 on MdHB7L‐mediated transcriptional regulation of downstream target genes, including MdCBF1/3, *MdPRX52/53*, *MdRD26*, MdPAP2, MdERDL6‐1/6‐7, and *MdERD5*. Fluorescence observation and relative LUC activity measurements showed that co‐expressing MdHOS1 significantly inhibited MdHB7L‐mediated transcriptional activation or inhibition of these genes' promoters (Figure 9F–I). Y2H assays identified the homeodomain (HD) of MdHB7L as crucial for its interaction with MdHOS1 (Figure 9J). Since the HD domain facilitates DNA binding in HD‐Zip TFs,^[^ ^54^, ^55^ ^]^ we examined whether MdHOS1 affects MdHB7L's promoter binding ability. In the promoter of each MdHB7L target gene, the binding site closest to the start codon was selected for EMSA assays. The addition of MdHOS1‐GST substantially decreased the band intensity of the MdHB7L‐probe complexes, indicating that MdHOS1 inhibited MdHB7L's binding ability (Figure 9K). These results suggest that besides mediating MdHB7L degradation, MdHOS1 also affects MdHB7L function at the transcriptional level.

## 3 Discussion

Due to global warming and the associated extreme weather events, plants are increasingly subjected to cold stress. HD‐Zip proteins are a class of TFs unique to plants, playing wide‐ranging roles in regulating plant growth, development, and responses to environmental stresses.^[^ ^24^, ^25^, ^26^ ^]^ However, their functions and regulatory mechanisms in cold stress responses remain unclear. This study identified the positive roles of MdHB7L in apple cold tolerance via both CBF‐dependent and CBF‐independent pathways. Furthermore, by analyzing the intricate competitive interactions among MdHB7L, MdICE1L, and MdHOS1 proteins, and the effects of these interactions on the transcriptional activity and protein stability of MdICE1L and MdHB7L, we elucidated the fine regulation of cold signaling mediated by the MdHB7L–MdICE1L–MdHOS1 module. This provides new insights into the dynamic cold response in plants.

### 3.1 MdHB7L Functions Positively in Cold Response via the CBF‐Dependent Pathway in both ICE1‐Dependent and ICE1‐Independent manners

ICE1, a well‐known positive regulator in cold response, directly activates the expression of CBFs and subsequently downstream CORs upon exposure to low temperatures.^[^ ^9^ ^]^ This triggers various physiological and biochemical adjustments, such as hormone synthesis, antioxidant enzyme activity, flavonoid metabolism, and accumulation of osmoregulatory substances, ultimately enhancing plant cold tolerance.^[^ ^1^, ^2^, ^6^ ^]^ Our study reveals that MdHB7L positively regulates cold tolerance in apple (Figure 1). Furthermore, we discovered an interaction between MdHB7L and MdICE1L, which was confirmed both in vivo and in vitro through various experiments (Figure 2A–D). These findings suggest that MdHB7L may participate in cold response by influencing MdICE1L's function. Previously, we identified MdCBF1 and MdCBF3 as direct target genes of MdICE1L.^[^ ^16^ ^]^ Therefore, we examined the effect of MdHB7L on MdICE1L's transcriptional regulatory activity. We found that co‐expressing MdHB7L significantly enhanced the binding and transcriptional activation of MdICE1L on MdCBF1/3 promoters (Figure 2E–I). These results indicate that MdHB7L promotes MdCBFs expression in an ICE1‐dependent manner.

Studies have shown that ICE1‐interacting proteins can regulate the expression of CBFs not only by influencing ICE1 activity but also by directly binding to their promoters. Examples in *Arabidopsis* include MYB15, MYB43, MYC67, and MYC70,^[^ ^21^, ^22^, ^56^ ^]^ and similar findings have been observed in apples, such as MdBBX37 and MdbHLH4.^[^ ^16^, ^20^ ^]^ The presence of multiple potential HD‐Zip binding sites in the MdCBF1/3 promoters led us to hypothesize that they may be direct targets of MdHB7L (Figure 3A). As expected, a series of assays confirmed that MdHB7L directly binds to these sites and activates the transcription of MdCBF1/3 under cold conditions (Figure 3B–G). Furthermore, mutating the core sequences of the bHLH TF potential binding sites in the MdCBF1/3 promoters demonstrated that MdHB7L‐mediated activation occurs independently of MdICE1L (Figure 3H), as well as other ICE1 homologs in apple, such as MdCIbHLH1/MdICE1.^[^ ^19^, ^20^ ^]^ These results suggest that MdHB7L can directly activate MdCBFs expression in an ICE1‐independent manner.

### 3.2 MdHB7L Positively Regulates Cold Tolerance via CBF‐Independent Pathways

Several TFs reportedly regulate plant cold responses through both CBF‐dependent and CBF‐independent pathways, such as BZR1 in *Arabidopsis*,^[^ ^57^ ^]^ and MdHY5, MdNAC104, and MdMYB88/124 in apples.^[^ ^6^, ^58^, ^59^ ^]^ HD‐Zip TFs have been shown to enhance plant resistance to adverse environments by regulating various physiological and biochemical changes, including antioxidant enzyme activity, osmoregulatory substance accumulation, and ion homeostasis.^[^ ^24^, ^25^, ^33^, ^34^, ^36^ ^]^ However, the molecular mechanisms underlying these processes remain unclear. To explore these mechanisms, we identified potential target genes of MdHB7L using ChIP‐seq (Figure 4). GO and KEGG pathway annotation revealed that these targets are primarily involved in hormone synthesis and signal transduction, as well as the accumulation of osmoregulatory substances and antioxidants (Figure 4E,F). This suggests that MdHB7L may also regulate cold tolerance through CBF‐independent pathways.

To identify target genes related to resistance regulation, we combined previously obtained transcriptomic data of MdHB7L‐OE plants^[^ ^36^ ^]^ with the ChIP‐seq data. Under strict screening thresholds, we identified two POD enzyme‐encoding genes, *MdPRX52*/*53* (Figure 5A,B; Table S6, Supporting Information). Further analysis confirmed the binding and transcriptional activation activity of MdHB7L on *MdPRX52/53* promoters. Additionally, we demonstrated the positive effects of MdHB7L on antioxidant enzyme activity and ROS scavenging under cold stress (Figure 5C–K). In addition to antioxidant enzymes, the accumulation of flavonoids and osmoregulatory substances like anthocyanins, soluble sugars and proline, plays a crucial role in plant cold tolerance.^[^ ^1^, ^6^ ^]^ Under cold conditions, MdHB7L significantly promotes the accumulation of these substances (Figure 6D). To understand how MdHB7L regulates this accumulation, we slightly lowered the threshold for target gene screening in our multi‐omics analysis. This approach revealed key genes involved in the biosynthesis or metabolism of these substances. Notably, we identified MdPAP2, which promotes anthocyanin accumulation,^[^ ^48^, ^49^, ^60^, ^61^ ^]^ MdERDL6s, which enhances soluble sugar accumulation,^[^ ^50^, ^62^ ^]^ and *MdERD5*, which mediates proline metabolism^[^ ^51^, ^52^ ^]^ (Figure 6A,B; Table S9, Supporting Information). Furthermore, our study demonstrated that MdHB7L could directly bind to the promoters of these genes. It activates the expression of MdPAP2 and MdERDL6s but inhibits the expression of *MdERD5* (Figure 6C–I). These findings suggest that MdHB7L enhances apple cold tolerance via a CBF‐independent pathway. Given the importance of these antioxidant enzymes and osmoregulatory substances in plant resistance to various abiotic stresses, this regulatory model might also apply to a broader range of resistance mechanisms, such as MdHB7L‐mediated drought and salt tolerance.^[^ ^25^, ^35^, ^36^ ^]^

### 3.3 MdHOS1 Mediates Ubiquitination and Degradation of MdICE1L and MdHB7L, Negatively Modulating Cold Tolerance

Post‐translational modifications, such as sumoylation, phosphorylation, and ubiquitination, play a significant role in regulating the activity and stability of TFs.^[^ ^1^, ^2^, ^10^, ^37^ ^]^ Studies have demonstrated that these modifications, including phosphorylation and ubiquitination, also affect HD‐Zip TFs.^[^ ^63^, ^64^, ^65^, ^66^ ^]^ In our study, we discovered that MdHB7L interacts with MdHOS1 (Figure 7A–D). In *Arabidopsis*, HOS1 is an E3 ubiquitin ligase that facilitates the ubiquitination of various TFs, including ICE1, leading to their degradation via the 26S proteasome pathway and negatively regulating cold response.^[^ ^2^, ^39^, ^42^ ^]^ We hypothesized that MdHOS1 might be involved in the MdHB7L‐mediated cold response by promoting MdHB7L degradation. Our findings confirmed that MdHOS1 directly mediates the ubiquitination and degradation of MdHB7L, with this process inhibited by cold treatment (Figure 8A–G). Furthermore, we identified the negative role of MdHOS1 in cold tolerance through detailed studies in transgenic *Arabidopsis* and apple calli (Figure S3, Supporting Information). Given the high sequence and functional similarity of HOS1 and ICE1 homologous genes in *Arabidopsis* and apple, we also investigated whether MdHOS1 influences MdICE1L degradation. As anticipated, MdHOS1 promotes MdICE1L degradation via the ubiquitin 26S pathway (Figure 8K–N). These results suggest that MdHOS1 negatively regulates cold tolerance by mediating the ubiquitination and degradation of both MdICE1L and MdHB7L.

Besides facilitating the ubiquitination of target proteins, HOS1 can also function as a cofactor, influencing TF activity or altering chromatin modifications in the promoter region of target genes, thereby affecting downstream gene transcription. For instance, HOS1 suppresses the transcriptional activity of PIF4 through direct interaction,^[^ ^43^, ^44^ ^]^ and serves as a chromatin remodeling factor for FLC regulation by interacting with histone deacetylase 6 (HDA6).^[^ ^67^ ^]^ Based on the MdHOS1–MdHB7L interaction, we explored whether MdHOS1 influences the regulation of target genes by MdHB7L. Functional analyses in transgenic calli and apple plants revealed that MdHOS1 weakens the cold tolerance enhanced by MdHB7L (Figure 9A–E). This is likely due to the negative impact of MdHOS1 on the expression of MdHB7L‐mediated target genes (Figure 9F–I). Y2H assays identified the HD domain of MdHB7L as the key region for the MdHB7L–MdHOS1 interaction (Figure 9J). Since the HD domain is crucial for DNA sequence binding by HD‐Zip TFs,^[^ ^54^, ^55^ ^]^ this finding suggests that MdHOS1 may inhibit the promoter binding ability of MdHB7L through direct interaction. The EMSAs results confirmed this hypothesis (Figure 9K). These results indicate that MdHOS1 is involved in the MdHB7L‐mediated cold response at both transcriptional and post‐translational levels. Considering the conserved HOS1‐ICE1 interaction across various plant species,^[^ ^39^, ^68^ ^]^ this dual regulatory role of HOS1 may also apply to ICE1 and could be relevant to a broader range of plant species.

### 3.4 MdHB7L Activates Cold Signaling in Different Ways During the Early and Late Stages of Cold Response, which is Related to Cold‐Induced MdICE1L Degredation

In *Arabidopsis*, CBFs are rapidly activated in response to cold, indicating that their upstream activators are already present.^[^ ^9^, ^69^ ^]^ Subsequent studies have found that ICE1 accumulates under normal conditions, allowing it to quickly activate CBF expression and initiate the early cold signaling at low temperatures.^[^ ^8^, ^9^, ^38^, ^39^ ^]^ On the other hand, low temperatures promptly induce HOS1 to migrate into the nucleus, leading to ICE1 degradation and subsequently terminating the ICE1‐mediated cold response.^[^ ^39^, ^70^ ^]^ Unlike the rapid degradation of ICE1, CBFs and their downstream CORs exhibit a more sustained upregulation pattern under cold stress, although not as intense as the initial ICE1‐mediated response.^[^ ^4^, ^9^, ^39^, ^40^, ^41^, ^42^ ^]^ This suggests the involvement of other TFs in activating CBFs expression after ICE1 degradation, thereby maintaining the plant's response to cold. Indeed, many TFs have been identified that directly activate *CBF* expression.^[^ ^1^, ^2^, ^5^ ^]^ However, the specific roles of these TFs and ICE1 in plant cold signaling are not yet fully understood.

In this study, MdHB7L was found to enhance the binding and transcriptional activation activity of MdICE1 on MdCBF promoters through interaction (Figure 2). However, unexpectedly, MdICE1L inhibited the direct binding of MdHB7L on the HD‐Zip TF binding motifs in MdCBF promoters (Figure 3G). Furthermore, by using mutated MdCBF promoters, we demonstrated that MdHB7L‐activated MdCBFs expression dependent on HD‐Zip TF binding motifs was significantly inhibited by MdICE1L (Figure 3H). These results indicate that the MdHB7L–MdICE1L complex activates MdCBFs experssion by binding to the MdICE1L‐binding sites, rather than the MdHB7L‐binding sites. In this case, the function of MdHB7L as a TF is inhibited by MdICE1L through interaction. These findings imply that in early cold response, when MdICE1L levels are high (Figure 8N), MdHB7L primarily acts as a cofactor to enhance MdICE1L‐mediated cold signaling, rather than as a TF to directly activate MdCBFs expression. Compared with MdHB7L, the stronger transcriptional activation activity and faster degradation rate of MdICE1L under cold also indicate that it should play a key role in early cold response (Figures 3I, 8N). Considering that MdICE1L may recruit MdHB7L as a cofactor through interaction, these findings also imply that the CBF‐dependent pathway, rather than the CBF‐independent pathway, may play a major role in MdHB7L‐mediated cold response in early stage of cold response in apple. On the other hand, this study identified pairwise interactions between MdICE1, MdHB7L, and MdHOS1 (Figure 7A–H), suggesting the existence of potential antagonistic interactions among these proteins. Notably, MdICE1L was found to interact preferentially with MdHOS1, effectively inhibiting the interaction between MdHB7L and MdHOS1 (Figure 7I–O). This preferential interaction likely elucidates the inhibitory effect of MdICE1L on MdHOS1‐mediated ubiquitination and degradation of MdHB7L (Figure 8H–L). Additionally, protein degradation assays demonstrated that MdHB7L degradation only began after MdICE1L protein levels had substantially decreased (Figure 8N). These results imply that in later stage of cold response, after a large amount of MdICE1L is degraded, excess MdHB7L will be able to act as a TF to directly regulate the expression of MdCBFs and other target genes, thereby sustaining the plant's response to cold stress. Given that MdHB7L has many direct target genes involved in cold response (Figure 3, 4, 5, 6), and its activation of MdCBF1/3 is not substantially stronger than other target genes (Figures 3F, 5G, and 6E), these findings also imply that in later stage of cold response, the CBF‐independent pathway may play a major role in MdHB7L‐mediated cold response, while the CBF‐dependent pathway assists in maintaining the overall cold response. Further studies are needed to verify this hypothesis.

**Figure 10:**
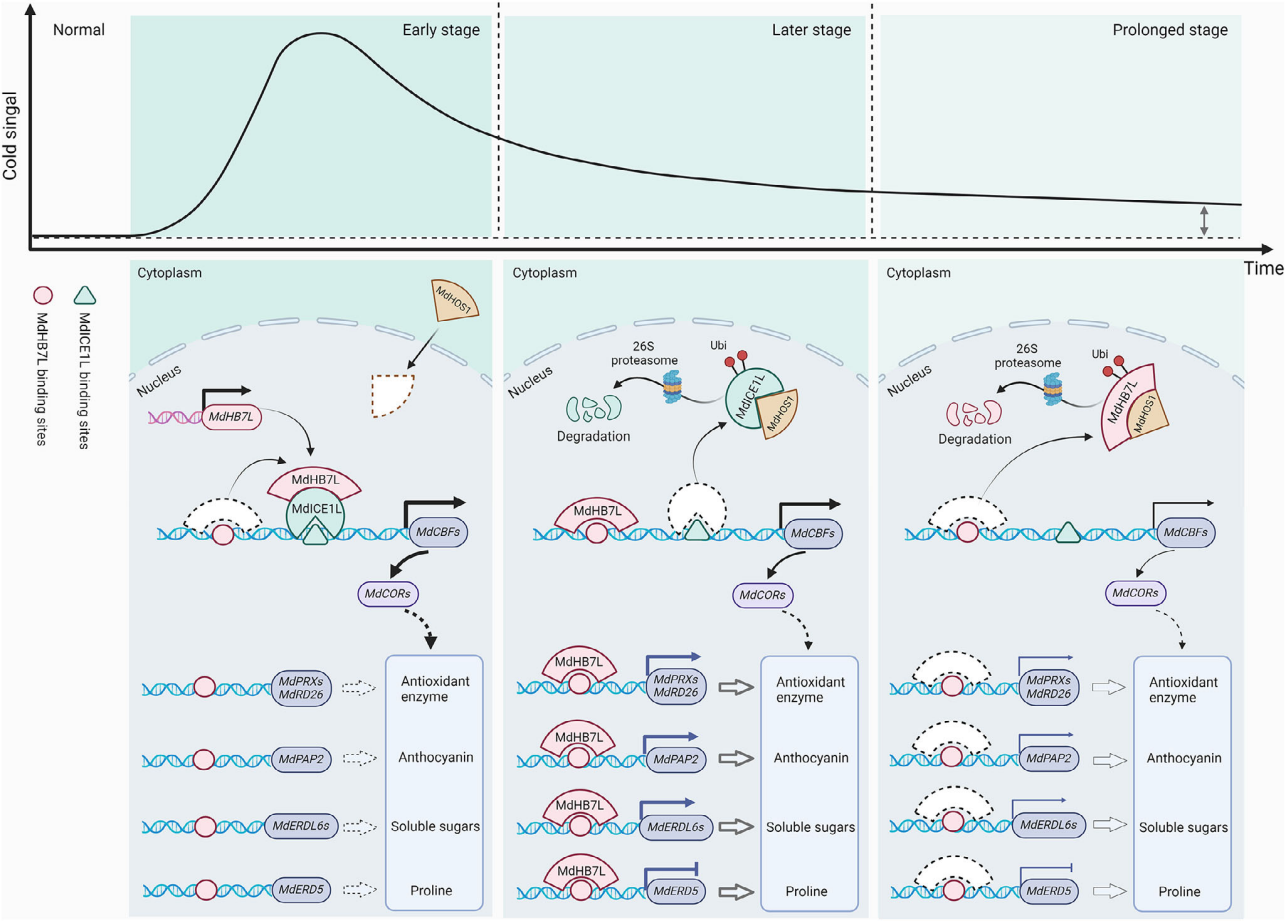
A proposed working model illustrating the dynamic response to cold stress mediated by the MdHB7L–MdICE1L–MdHOS1 module. Upon sudden exposure to cold stress, MdICE1L is rapidly activated and functions as a potent transcriptional activator, initiating early cold signaling by activating MdCBFs expression. During this process, MdICE1L recruits MdHB7L as a cofactor through direct interaction to enhance its own promoter binding and transcriptional activation activity. In response to cold, MdHOS1 gradually translocates into the nucleus, where it interacts with MdICE1L and mediates its ubiquitination and degradation, resulting in a rapid and sustained reduction in MdICE1L protein level. This process is accompanied by the release and accumulation of MdHB7L. The accumulated MdHB7L directly binds to the promoters of several target genes, including MdCBF1/3, *MdPRX52/53*, *MdRD26*, MdPAP2, MdERDL6‐1/6‐7, and *MdERD5*, regulating their transcription, and thus enhancing plant cold tolerance through both CBF‐dependent and CBF‐independent pathways. As MdICE1L levels continue to decrease, excess MdHOS1 interacts with MdHB7L, inhibiting its binding to target gene promoters and promoting its ubiquitination and degradation, thereby suppressing MdHB7L‐mediated cold signaling.

Based on previous studies and these findings, we propose a regulatory model wherein the MdHB7L–MdICE1L–MdHOS1 module fine‐tunes the cold response through both CBF‐dependent and CBF‐independent pathways (**Figure** **10**). Upon exposure to cold stress, abundant MdICE1L utilizes MdHB7L as a cofactor to facilitate the transcriptional activation of MdCBFs, thereby activating early cold signaling rapidly and strongly. Subsequently, MdICE1L is degraded by MdHOS1 in response to cold stimuli. Meanwhile, MdHB7L is released and accumulates. It then acts as a TF to directly regulate the expression of MdCBFs and other target genes, thereby sustaining the plant's response to cold environments. As MdICE1L levels continue to decrease under cold, excess MdHOS1 interacts with MdHB7L and mediates its degradation, thereby inhibiting MdHB7L‐mediated cold signaling. Our study uncovers distinct temporal and activity‐related roles of MdICE1L and MdHB7L in cold signaling pathways, providing new insights into the intricate mechanisms of the dynamic cold response in plants.

## 4 Experimental Section

### Plant Materials and Growth Conditions

Plant materials used in this study include ′GL‐3″ apple plants,^[^ ^71^ ^]^ apple calli (“Orin”), and A. thaliana seedlings (*Col‐0*). MdHB7L‐OE/RNAi transgenic apple plants and MdHB7L‐Myc transgenic calli were obtained from previous studies.^[^ ^25^, ^35^ ^]^ Apple plants were grown at 25 °C under long‐day (LD) conditions (16 h light/8 h dark). “Orin” calli were grown on MS medium with 1.5 mg L^−1^ 2,4‐D and 0.4 mg L^−1^ 6‐BA (6‐benzyl aminopurine) in the dark at 25 °C, unless otherwise stated. *Arabidopsis* seedlings were grown at 23 °C under short‐day (SD) conditions (8 h light/16 h dark) before cold treatment.

### Cold Treatment

Both *Arabidopsis* and apple plants with uniform growth were subjected to two types of freezing treatments: with cold acclimation (CA; 4 °C for 12 h in the dark) or without cold acclimation (NA). A step‐by‐step cooling method was used, as described previously.^[^ ^6^, ^16^ ^]^

Tissue‐cultured MdHB7L transgenic and GL‐3 (wild type; WT) apple plants were transferred to rooting medium (MS medium with 0.5 mg L^−1^ IAA (3‐Indoleacetic acid) and 0.5 mg/L IBA (Indolebutyric acid). After 35 d, rooted plants were transplanted into pots with a mixture of nutrient soil and perlite (1:1, v/v) and grown in a growth chamber under LD conditions for 2 months. Plants in the NA group were treated at −6 °C for 4 h, while plants in the CA group were treated at −8 °°C for 4 h after cold acclimation. Following freezing treatment, plants were placed at 4 °C for 12 h in the dark for recovery, then transferred to LD conditions at 25 °C. After 4 d, photographs were taken, and physiological parameters were measured. Each group had three biological replicates, with 20 plants per line (OE#1, OE#3, GL‐3, RNAi#1, and RNAi#3) in each replicate.

For the freezing treatment of apple plants cultured in MS medium, transgenic plants were subjected to −10 °C for 2 h without prior cold acclimation. Three biological replicates were performed, with 32 transgenic plants per genotype (OE/RNAi) in each replicate. For leaf relative electrolyte leakage (REL) assays, leaves of apple plants were punched into leaf discs with a diameter of 6 mm. Four leaf discs from two plants were placed in a test tube containing 500 µL of deionized water. The tubes were then placed into a low‐temperature water bath cycle instrument (Thermo Fisher PC200‐A40) for freezing treatment, referring to the methods previously described,^[^ ^59^, ^72^ ^]^ with some modification. The freezing treatments were carried out using a step‐by‐step cooling method, starting from 0 °C and decreasing the temperature at a rate of 2 °C h^−1^ until −6 °C, then hold at −6 °C for 2 h. Six biological replicates were performed, with three tubes per genotype in each replicate.

For the cold treatment of *Arabidopsis* plants, 6‐week‐old plantlets were divided into NA (−6 °C, 3 h) and CA (−8 °C, 4 h) treatment groups. The post‐freezing treatments were consistent with those of apple plants. Each group contained three biological replicates, with 36 plants per line in each replicate.

For apple calli, WT and transgenic calli blocks (0.1 g) of similar growth status were pre‐cultured on new medium plates for 5 d under normal conditions (25 °C, dark). These plates were then subjected to 4 °C under dark conditions for cold treatment. After the specified treatment time, images were taken and the fresh weight was measured. Each replicate consisted of three plates, with a total of three biological replicates.

### Vector Construction and Genetic Transformation

The full‐length coding sequences (CDSs) of MdHOS1, GFP (green fluorescent protein), and a gene‐specific fragment of MdHOS1 (355 bp) were inserted into the pCambia2300‐3HA and pK7GWIWG2D RNAi vector (carrying a GFP marker for transformant selection). These vectors were transformed into apple calli using the *Agrobacterium tumefaciens* EHA105‐mediated method^[^ ^73^ ^]^ to obtain MdHOS1‐HA, *MdHOS1*‐RNAi, and GFP–HA transgenic calli. These vectors were also transformed into MdHB7L‐Myc calli to obtain MdHOS1‐HA+ MdHB7L‐Myc, MdHOS1‐RNAi+ MdHB7L‐Myc, and GFP–HA + MdHB7L‐Myc double transgenic calli. The MdICE1L‐Flag + MdHOS1‐HA and MdICE1L‐Flag + MdHB7L‐Myc double transgenic calli were obtained using the same method. Triple transgenic calli were obtained using a vacuum‐based transient transformation method^[^ ^74^ ^]^ with these double transgenic calli as the background.

MdHOS1‐GFP, MdHOS1‐RNAi, and an empty vector (GFP tag) were instantaneously transformed into MdHB7L transgenic (OE#3) apple plants using a vacuum‐based transient transformation method^[^ ^75^ ^]^ to obtain MdHB7L + MdHOS1 double transgenic plants. After 3 d of culture, some of the transiently transformed plants were randomly selected for western‐blot analysis. MdICE1L‐RNAi, MdHOS1‐RNAi, MdHB7L‐RNAi + MdICE1L‐RNAi, and MdHB7L‐RNAi + MdICE1L‐RNAi + MdHOS1‐RNAi transgenic plants were created in the background of WT and MdHB7L‐RNAi (RNAi#1) transgenic plants, respectively, by the same method. After 3 d of culture, some leaves of the transiently transformed plants were randomly selected for gene expression analysis.

The MdHB7L promoter fragment (1500 bp upstream of the start codon) was cloned into the pCambia1301‐GUS vector to replace the 35S promoter. Then, the constructed pMdHB7L‐GUS vector was transformed into *Arabidopsis* using the A. tumefaciens GV3101‐mediated floral dip method.^[^ ^73^ ^]^ Positive transformants were identified using qRT‐PCR and western blot analyses. The sequences of the primers used for vector construction were listed in Table S10 (Supporting Information).

### Yeast‐One‐Hybrid (Y1H) Assay

Various fragments of MdCBF1 and MdCBF3 promoters (100 bp in length), each containing the typical HD‐Zip transcription factor binding motif, were cloned into the pLacZi vector. The CDS of MdHB7L was cloned into the pB42AD vector. These constructs were transformed into the EGY48 yeast strain in specified combinations, with empty pB42AD and N (randomly selected promoter fragments without the binding motif) as negative controls. The assays were conducted according to previously described methods.^[^ ^76^, ^77^ ^]^ The binding of MdHB7L to the specific promoter fragments of MdCBF1/3 was indicated by the blue color of the transformed yeast.

### Dual‐LUC Assay

The CDS of MdHB7L (or MdICE1L) was cloned into the pGreenII 62‐SK vector. The promoter sequences of target genes, with normal and mutated elements (Hbmut, bHLHmut), were inserted into the pGreenII 0800‐LUC vector. These vectors were transiently expressed in *Nicotiana benthamiana* leaves using specified combinations via the A. tumefaciens GV3101‐mediated method. After 2 d of culture under normal conditions (25 °C, LD), plants were transferred to 4 °C for 6 h. Then, the areas surrounding the injection site were collected for luminescence detection using the Dual Luciferase Reporter Gene Assay Kit (Yeasen Biotechnology Co., Ltd., Shanghai, China). LUC fluorescence was captured using a Lumazone Pylon 2048B imaging system (Princeton, NJ, USA). Each biological replicate contained four leaves from two tobacco plants, with three replicates.

### Electrophoretic Mobility Shift Assay (EMSA)

The CDSs of MdHB7L and MdICE1L were cloned into the pET28a vector, and the CDS of MdHOS1 was cloned into the pGEX‐4T‐1 vector. These constructs were transformed into Escherichia coli BL21(DE3) for expression. The expressed proteins were purified using HIS‐tag and GST‐tag purification resins (Beyotime). EMSAs were performed using the Chemiluminescent EMSA Kit (Beyotime) according to the manufacturer's instructions. MdHOS1‐GST protein was added to the system to assess its impact on MdHB7L binding ability. The sequences of probes were listed in Table S10 (Supporting Information).

### ChIP‐qPCR Assay

MdICE1L‐Flag, MdHB7L‐Myc, MdICE1L‐Flag+MdHB7L‐Myc, and corresponding control transgenic calli (GFP‐Flag or GFP‐Myc) were treated at 4 °C for 6 h and then harvested for cross‐linking in formaldehyde solution. After sonication, chromatin extracted from these various transgenic calli was immunoprecipitated with (or without) anti‐Flag/Myc antibody (Yeasen, Shanghai, China). The relative enrichment of promoter fragments was determined by qPCR, with the enrichment of control samples (empty vector) serving as the reference and set to 1.0. The experiment consisted of three biological replicates, each with four technical replicates. Primer sequences were listed in Table S10 (Supporting Information).

### ChIP‐seq

The ChIP‐seq assay was performed by Wuhan SeqHealth Tec Co. (Wuhan, China), following previously described methods.^[^ ^59^, ^78^ ^]^ In summary, MdHB7L‐Myc transgenic calli treated at 4 °C for 6 h were cross‐linked and immunoprecipitated using anti‐Myc antibody. Samples not subjected to immunoprecipitation served as controls (Input). The DNA fragments were then sequenced using paired‐end (PE) technology on the Illumina TruSeq platform. Quality‐filtered reads were aligned to the apple reference genome (GDDH13; https://www.rosaceae.org/) using Bowtie2 software, and MACS2 was used for peak calling.

### Yeast‐Two/Three‐Hybrid (Y2H/Y3H) assays

The CDSs or truncated fragments of MdHB7L, MdHOS1, and MdICE1L were inserted into the pGBKT7 and pGADT7 vectors, respectively. Truncated fragments of MdHOS1 (MdHOS1‐C150) and ***MdHB7L*** (or *MdICE1L*) were inserted into the MCSI and MCSII sites of the pBridge vector to generate the pBridge‐MdHOS1(‐C150), pBridge‐MdHOS1(‐C150)‐MdHB7L, and pBridge‐MdHOS1(‐C150)‐MdICE1L vectors. The recombinant vectors were co‐transformed into the yeast strain Y2H‐Gold in specific combinations. Positive transformants that grew on SD‐Leu/‐Trp (DDO) or SD‐Leu/‐Trp/‐Met medium were transferred to a screening medium (Y2H: SD‐Leu/‐Trp/‐His/‐Ade, QDO; Y3H: SD‐Leu/‐Trp/‐His/‐Ade/‐Met) to test for possible interactions. For interaction activity comparison, yeast transformants were cultured in YPDA medium until OD_600_ = 0.6. Then, 1 mL of yeast culture were centrifuged and washed thrice with 0.9% NaCl solution. Aliquots of 10 µL each 10‐fold serial dilution (10^1^, 10^2^, 10^3^) were then spotted onto new plates.

### Co‐IP Assay

The MdHOS1‐HA, MdICE1L‐Flag, and MdHB7L‐Myc/‐Flag vectors were transiently co‐expressed in N. benthamiana leaves in specified combinations. After 3 days of culture under standard conditions, total protein was extracted from the leaves and incubated with anti‐HA/Flag/Myc magnetic beads (Beyotime) at 4 °C overnight. The eluted proteins were detected using anti‐HA/Flag/Myc antibodies (Yeasen).

### Pull Down Assay

MdHB7L–MBP (pMAL‐c5x) recombinant proteins were purified using MBP‐tag purification resin (Beyotime). MdHOS1–GST, MdHB7L–HIS/MBP, and MdICE1L–HIS proteins were co‐incubated overnight at 4 °C in designated combinations. Pull‐down assays were performed with anti‐HIS/GST magnetic beads following the manufacturer's instructions (Beyotime). The eluted solutions were then analyzed using anti‐HIS/GST/MBP antibodies (Beyotime).

### LUC Complementation Imaging (Split‐LUC) Assay

The CDSs of MdHB7L, MdICE1L, and MdHOS1 were cloned into the pRI101‐cLUC and pRI101‐nLUC vectors, respectively. These vectors were co‐injected into tobacco leaves in specific combinations for transient expression. The plants were cultured for two days in a growth chamber under LD conditions at 25 °C. The infiltrated leaves were then collected for fluorescence imaging using a Lumazone Pylon 2048B system (Princeton, NJ, USA). Combinations containing empty cLUC or nLUC vectors were used as negative controls.

### Protein Degradation and Ubiquitination Assays

To assess the effects of cold on MdHB7L protein stability, MdHB7L‐Myc transgenic calli were treated either at room temperature (25 °C; control) or at 4 °C in MS medium with 250 µm cycloheximide (CHX), with or without 100 µm MG132. Samples were collected at specified time points for total protein extraction, and MdHB7L‐Myc protein levels were analyzed using an anti‐Myc antibody.

To determine the influence of MdHOS1/MdICE1L on MdHB7L stability in vitro, various MdHOS1 and MdHOS1 + MdICE1L transgenic calli were generated, and MdHB7L‐HIS fusion protein was prepared for cell‐free degradation assays as described previously.^[^ ^79^, ^80^ ^]^ Briefly, total proteins were extracted from these transgenic calli and incubated with purified MdHB7L‐HIS protein at 22 °C. Samples were collected at specified intervals and analyzed with an anti‐HIS antibody. For in vivo degradation assays, transgenic calli were treated at 4 °C in MS medium with 250 µm CHX, with or without 100 µm MG132. Samples were taken at specified time points for protein extraction and Western blotting.

For in vivo ubiquitination detection, protein extracts from transgenic calli treated at 4 °C for specified times (with CHX/MG132) were immunoprecipitated using anti‐Myc/anti‐Flag magnetic beads. The eluted proteins were then detected using anti‐Myc/anti‐Flag and anti‐Ubi antibodies (Cell Signaling Technology, USA). For in vitro ubiquitination detection, assays were performed as described previously.^[^ ^80^ ^]^ Briefly, MdHB7L‐HIS was incubated with MdHOS1‐GST in an incubation buffer at 4 °C for 10 h with agitation. Ubiquitinated proteins were detected using anti‐HIS and anti‐Ubi antibodies.

To investigate the effects of MdHOS1 on the degradation and ubiquitination of MdHB7L and MdICE1L in vivo using the LUC system,^[^ ^53^ ^]^ the CDSs of MdHB7L and MdICE1L were cloned into the pRI101‐LUC vector (driven by the CaMV35S promoter). These constructs, along with MdHOS1‐HA, were infiltrated into N. benthamiana leaves in specified combinations for transient expression. After 2 days of culture under normal conditions, the plants were treated at 4 °C for 6 h, with their leaves sprayed with 250 µm CHX (±100 µm MG132). The leaf luminescence was captured using a Lumazone Pylon 2048B imaging system.

### Physiological Parameter Measurements and Histochemical Staining

Leaf relative electrolyte leakage (REL) and total chlorophyll content were measured as described previously.^[^ ^6^, ^16^, ^25^ ^]^ The levels of H_2_O_2_, O_2_^−^, soluble sugar, and proline, along with antioxidant enzyme activities (SOD, POD, CAT, and total antioxidant capacity) in apple leaves, were measured using kits following the manufacturer's instructions (Comin Biotechnology, Suzhou, China). DAB and NBT staining of apple leaves was performed to observe H_2_O_2_ and O_2_^−^ accumulation, respectively, according to the previously described method.^[^ ^6^, ^25^ ^]^ For GUS staining and activity measurement, 7‐day‐old transgenic *Arabidopsis* seedlings were treated at 4 °C for 8 h. Histochemical staining and GUS activity assays were carried out using kits according to the manufacturer's instructions (Coolaber Science & Technology Co., Ltd., Beijing, China), with three biological replicates of nine plants each.

### RNA Extraction and RT‐qPCR

Total RNA was extracted from various plant materials using the RNAprep Pure Plant Kit (Tiangen, Beijing, China) following the manufacturer's protocol. Single‐stranded DNA synthesis was then conducted via reverse transcription using the PrimeScript RT Reagent Kit (TaKaRa, Shiga, Japan). Reverse transcription qPCR (RT‐qPCR) analysis was performed as described previously, with MdMDH serving as the reference gene.^[^ ^6^, ^16^, ^81^ ^]^

### Statistical Analysis

Data analysis was conducted using SPSS 22.0 software (IBM, Chicago, IL, USA). The significance of differences between means was assessed using one‐way ANOVA, Duncan's test or Student's *t*‐test (*p* < 0.05).

### Accession Numbers

The apple sequences data used in this study could be found in the Genome Database for Rosaceae (GDR; https://www.rosaceae.org; Malus × domestica GDDH13 v1.1) data libraries under accession numbers MdHB7L (MD01G1226600), MdICE1L (MD09G1003800), MdHOS1 (MD04G1060900), MdCBF1 (MD07G1262900), MdCBF3 (MD01G1196100), MdPRX52 (MD04G1170900), MdPRX53 (MD03G1013600), *MdRD26* (MD15G1079400), MdPAP2 (MD17G1261000), MdERDL6‐1 (MD15G1026400), MdERDL6‐7 (MD13G1175900), *MdERD5* (MD17G1070700).

## Conflict of Interest

The authors declare no conflict of interest.

## Author Contributions

J.Y. and N.L. contributed equally to this work. J.Y., K.M., and F.M. designed and supervised the experiment. J.Y., N.L., M.L., R.Y., L.Q., K.W., and S.Z. conducted experiments and analyzed the data. J.Y., and N.L. wrote the manuscript with inputs from all other authors. J.Y., K.M., and F.M. revised the manuscript and provided funding support. All authors read and approved the final manuscript.

## Supporting information



Supporting Information

Supplemental Table 1

Supplemental Table 2

Supplemental Table 3

Supplemental Table 4

Supplemental Table 5

Supplemental Table 6

Supplemental Table 7

Supplemental Table 8

Supplemental Table 9

Supplemental Table 10

## Data Availability

The data that support the findings of this study are available in the supplementary material of this article.
