# Supplementary material for: The MdHB7L–MdICE1L–MdHOS1 Module Fine‐Tunes Apple Cold Response via CBF‐Dependent and CBF‐Independent Pathways
Source: Adv Sci (Weinh). 2025 Apr 26;12(25):2501524. doi: 10.1002/advs.202501524 (PMC12224988; doi:10.1002/advs.202501524)
Supplement: Supplementary file 1 — Supporting Information [file ADVS-12-2501524-s008.pdf]

ADVANCED SCIENCE
Open Access

Supporting Information

for *Adv. Sci.*, DOI 10.1002/advs.202501524

The MdHB7L–MdICE1L–MdHOS1 Module Fine-Tunes Apple Cold Response via CBF-Dependent and CBF-Independent Pathways

Jie Yang, Na Li, Ming Li, Ran Yi, Lina Qiu, Kangning Wang, Shuang Zhao, Fengwang Ma and Ke Mao**

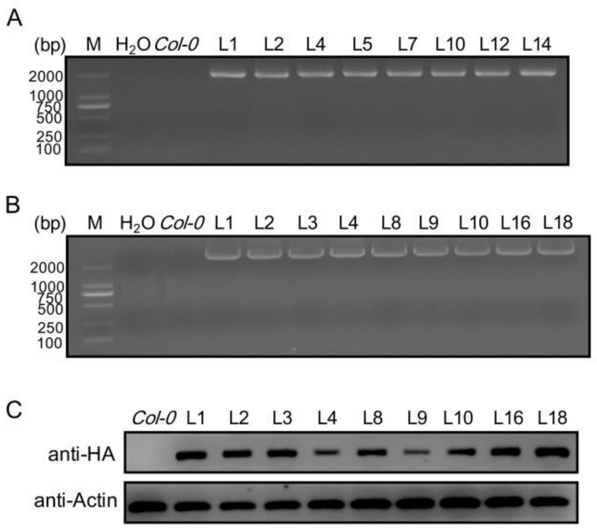

Supplementary Figure S1. Identification of *pMdHB7L*::GUS and *MdHOS1* transgenic *Arabidopsis* seedlings.

(A-B) Genomic PCR analysis of *pMdHB7L*::GUS (A) and *MdHOS1* (B) transgenic *Arabidopsis* seedlings. **(C)** Detection of MdHOS1-HA protein levels in different transgenic lines.

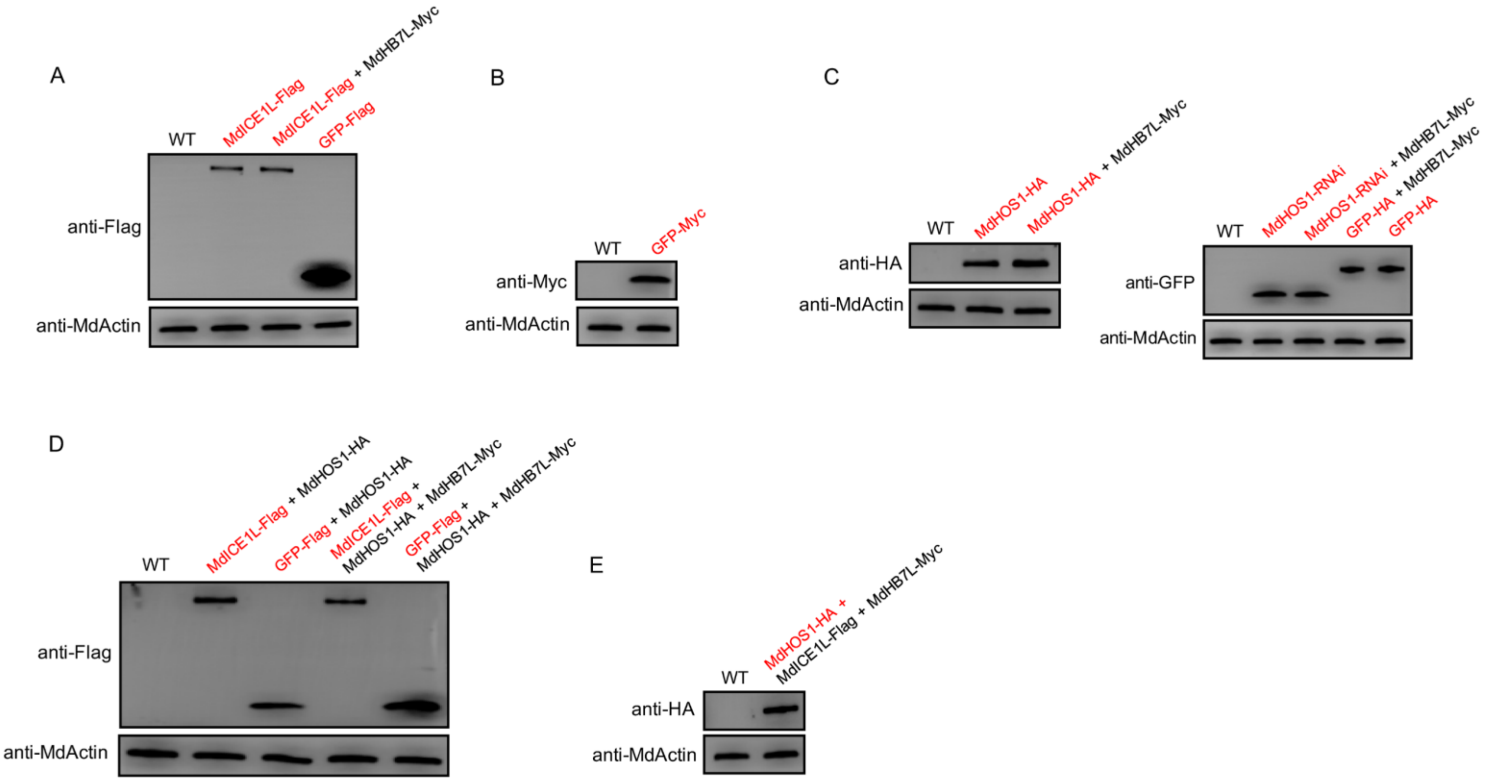

Supplementary Figure S2. Identification of various transgenic apple calli by western blot.

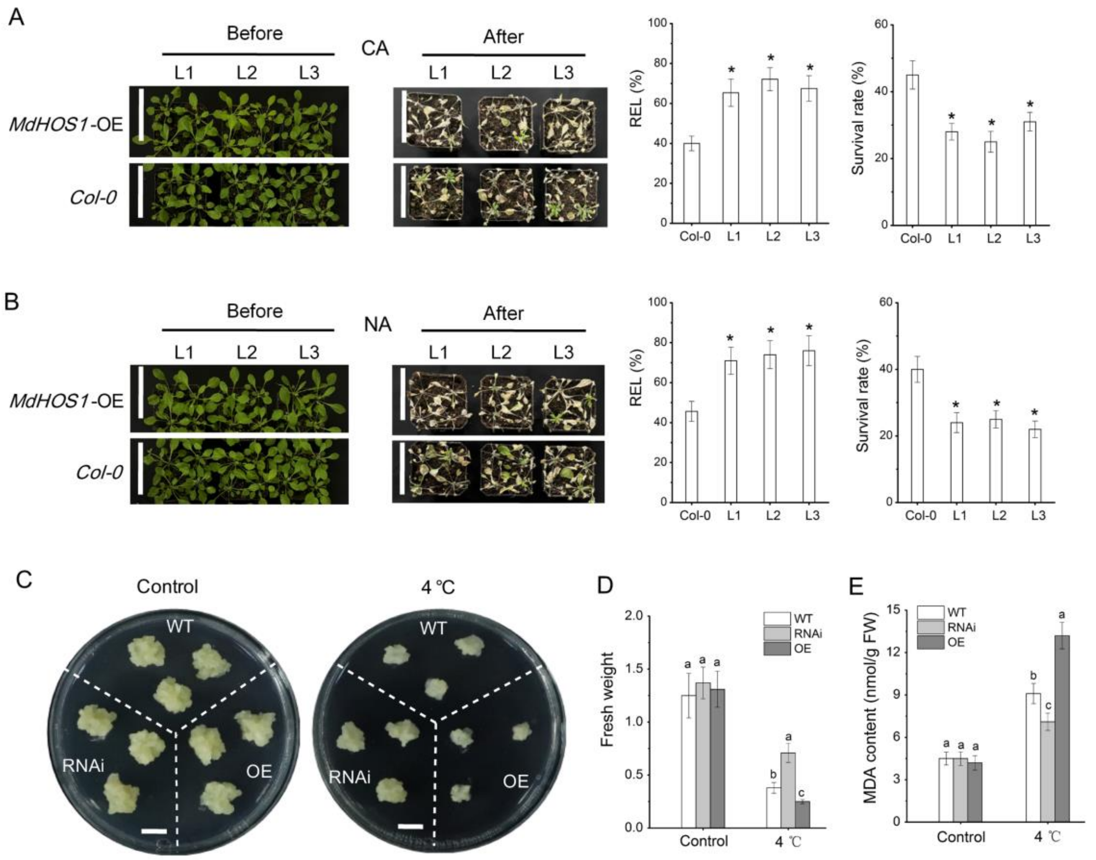

Supplementary Figure S3. *MdHOS1* negatively regulates cold tolerance in transgenic *Arabidopsis* and apple.

(A-B) Phenotypic observations, leaf relative electrolyte leakage (REL), and plant survival rate following freezing treatment. Scale bars, 7 cm. **(C-E)** Growth phenotypes (C), fresh weight (D), and malondialdehyde (MDA) content (E) in WT and *MdHOS1*-OE(HA)/RNAi transgenic calli. Scale bars, 1 cm. Error bars represent SD based on three biological replicates. Asterisks denote values significantly different from control ($P < 0.05$, Student's t-test). Different letters indicate significant differences at $P < 0.05$, as determined by one-way ANOVA and Duncan's tests.

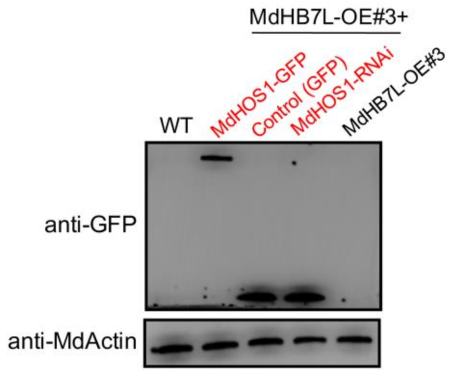

Supplementary Figure S4. Identification of *MdHB7L+MdHOS1* double transgenic apple plants through Western blotting. The transgenic line OE(#3) with high *MdHB7L* expression[35,36], served as the background for *MdHOS1*-GFP, control, and *MdHOS1*-RNAi vector transgenes.

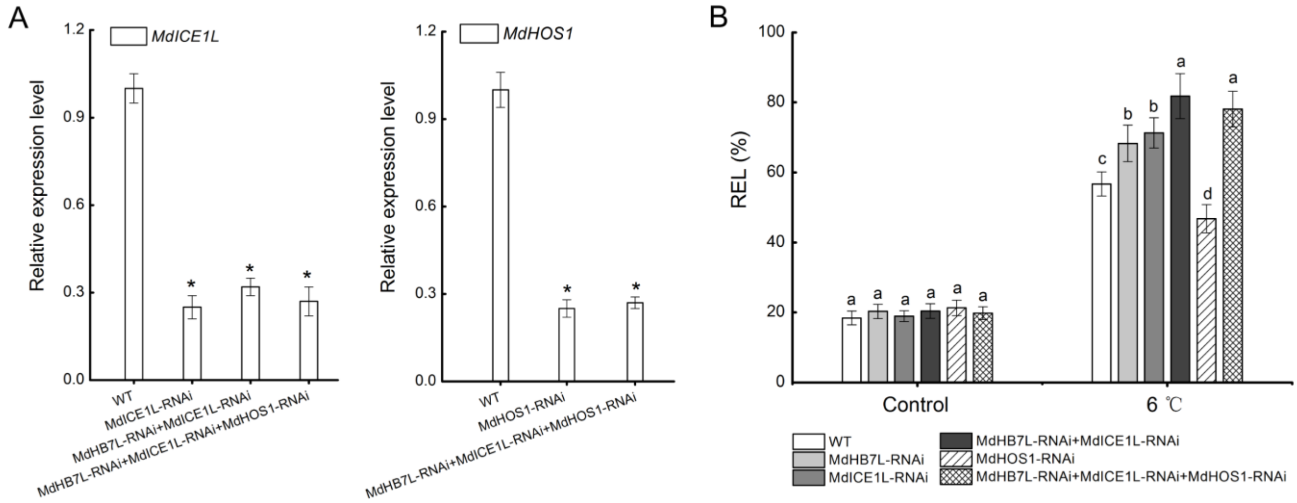

Supplementary Figure S5. The regulatory role of MdHOS1 in apple cold response depends on MdHB7L and MdICE1L.

(A) Identification of gene expression of *MdICE1L* and *MdHOS1* in leaves of transgenic plants. *MdICE1L*-RNAi and *MdHOS1*-RNAi transgenic lines were created in the background of WT plants, while *MdHB7L*-RNAi + *MdICE1L*-RNAi double transgenic line and *MdHB7L*-RNAi + *MdICE1L*-RNAi + *MdHOS1*-RNAi triple transgenic line were created in the background of *MdHB7L*-RNAi transgenic plants. Error bars represent SD based on three biological replicates. Asterisks denote values significantly different from control ($P < 0.05$, Student's *t*-test). **(B)** Freezing tolerance of various transgenic plants determined by leaf REL assays. Error bars represent SD based on six biological replicates. Different letters denote significant differences at $P < 0.05$ (Duncan's test).
